# Bone marrow Tregs mediate stromal cell function and support hematopoiesis via IL-10

**DOI:** 10.1172/jci.insight.135681

**Published:** 2020-11-19

**Authors:** Virginia Camacho, Victoria R. Matkins, Sweta B. Patel, Jeremie M. Lever, Zhengqin Yang, Li Ying, Ashley E. Landuyt, Emma C. Dean, James F. George, Henry Yang, Paul Brent Ferrell, Craig L. Maynard, Casey T. Weaver, Heth R. Turnquist, Robert S. Welner

**Affiliations:** 1 Division of Hematology-Oncology,; 2 Nephrology Research and Training Center, Division of Nephrology, Department of Medicine, and; 3 Division of Cardiothoracic Surgery, Department of Surgery, The University of Alabama at Birmingham, Birmingham, Alabama, USA.; 4 Cancer Science Institute of Singapore & Department of Biochemistry, National University of Singapore, Singapore.; 5 Department of Pathology, University of Alabama at Birmingham, Birmingham, Alabama, USA.; 6 Division of Hematology/Oncology, Vanderbilt University Medical Center, Nashville, Tennessee, USA.; 7 Department of Surgery, University of Pittsburgh, Pittsburgh, Pennsylvania, USA.; 8 Department of Immunology, University of Pittsburgh School of Medicine, Pittsburgh, Pennsylvania, USA.

**Keywords:** Hematology, Immunology, Bone marrow, Hematopoietic stem cells, T cells

## Abstract

The nonimmune roles of Tregs have been described in various tissues, including the BM. In this study, we comprehensively phenotyped marrow Tregs, elucidating their key features and tissue-specific functions. We show that marrow Tregs are migratory and home back to the marrow. For trafficking, marrow Tregs use S1P gradients, and disruption of this axis allows for specific targeting of the marrow Treg pool. Following Treg depletion, the function and phenotype of both mesenchymal stromal cells (MSCs) and hematopoietic stem cells (HSCs) was impaired. Transplantation also revealed that a Treg-depleted niche has a reduced capacity to support hematopoiesis. Finally, we found that marrow Tregs are high producers of IL-10 and that Treg-secreted IL-10 has direct effects on MSC function. This is the first report to our knowledge revealing that Treg-secreted IL-10 is necessary for stromal cell maintenance, and our work outlines an alternative mechanism by which this cytokine regulates hematopoiesis.

## Abstract 2

Bone marrow Tregs regulate stromal cell function to maintain hematopoiesis through IL-10 and provide a potential therapeutic avenue for stromal manipulation.

## Introduction

Tregs are required for the preservation of immunological homeostasis. Recent work has shown that Tregs also reside within specific tissues and contribute to nonimmune regulation of their local microenvironments (1–6). While the crosstalk between Tregs and other immune cells has long been an active area of investigation, there is a critical gap in knowledge defining how Tregs interact with nonhematopoietic cells and how these Treg properties may be exploited to target nonhematopoietic populations within tissue microenvironments.

Tregs have specialized roles in tissue regeneration, oral tolerance, and metabolic responses reflecting the functional and molecular heterogeneity of these cells (1, 7). These “tissue Tregs” are enriched at various sites, including skeletal muscle, epithelium, lamina propria, and adipose tissue (3, 4, 6, 8–11). Importantly, they differ from peripheral Tregs with respect to cytokine production, transcriptome profile, and metabolic capacities. In the BM, Tregs are found at a significantly higher ratio than in other lymphoid tissues (30%–40% of CD4^+^ T cells) (12, 13). In vivo imaging of BM has demonstrated that Tregs cluster proximal to hematopoietic stem cells (HSCs) and that adenosine generated by Tregs maintains HSC quiescence and can promote allo-HSC engraftment (14, 15). Additionally, Tregs have been shown to play a critical role in improving graft versus host disease (GVHD) and recovery following BM transplantation (16–18). These observations indicate that Tregs are active regulators of homeostasis within the BM.

Nonhematopoietic components of the BM and the mechanisms by which they maintain hematopoiesis have been extensively studied (19–23). HSCs are kept in balance by a dynamic milieu of cellular interactions within specialized microenvironments known as niches (24–31). This network of nonhematopoietic cells is loosely referred to as the stroma, and it encompasses a complex system of specialized cells. Among these are mesenchymal stromal cells (MSCs), a stromal cell with multilineage differentiation capacity to bone, fat, and cartilage (27, 30, 32–34). Many studies have underscored a role for stromal regulation of HSCs. However, little is known about how T cells regulate the stromal niche and facilitate these functions. This study examines Treg regulation of the BM microenvironment, with a direct focus on maintenance of MSCs.

Here, we outline the characteristics that define BM Tregs. We propose that marrow Tregs represent a specialized tissue Treg that actively regulates and conditions the stromal niche. In this study, we used multiple clinically validated immunomodulatory agents to manipulate the marrow Treg pool and assessed these effects on the BM microenvironment. Our results provide mechanistic insight into Treg regulation of MSCs, and we identify IL-10 signaling as highly relevant to this process. Importantly, our work provides a foundation for the further investigations into cell-based Treg therapy, specifically targeted at enhancing stromal cell function.

## Results

### Phenotypic markers distinguish peripheral and BM Tregs.

**Figure 1:**
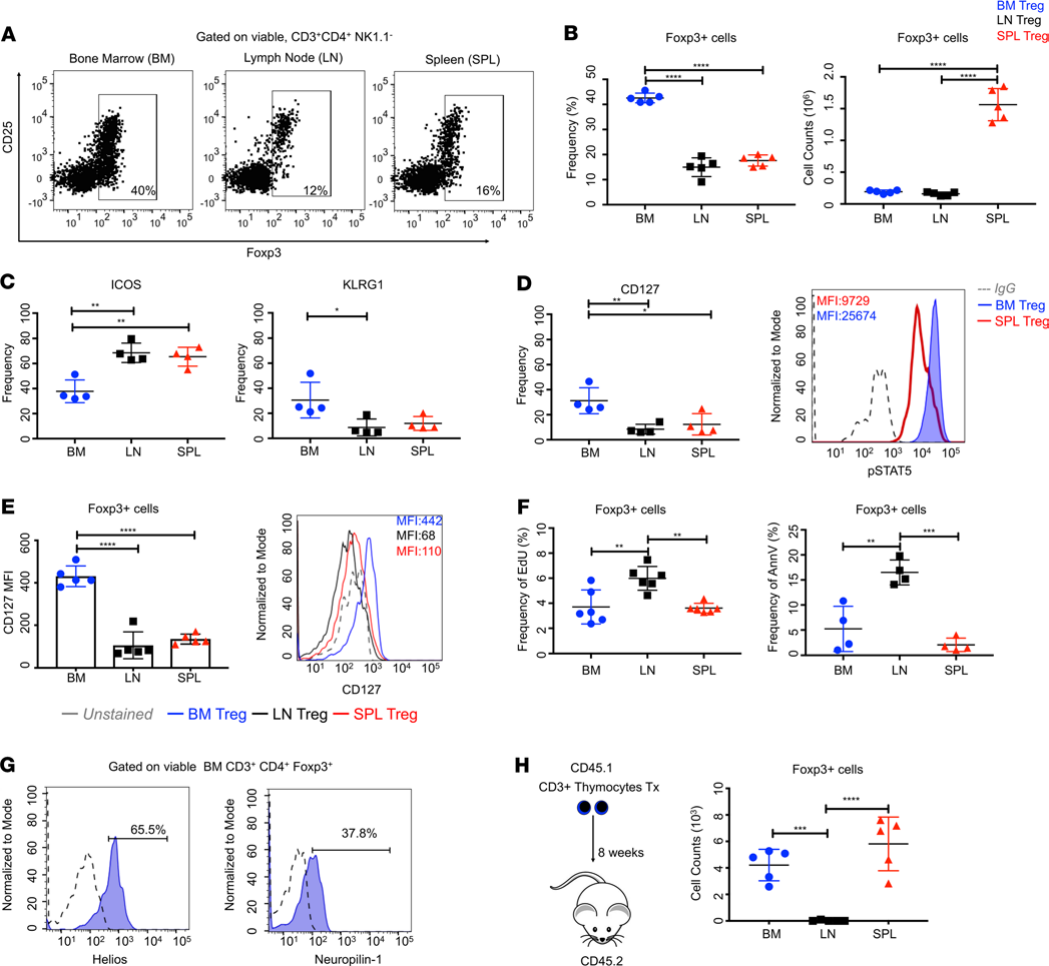
Profiling of bone marrow Tregs. (**A**) Representative plots of Foxp3^+^ cells. (**B**) Frequencies and absolute counts of Foxp3^+^ cells; *n* = 5 animals. (**C**) Frequencies of ICOS^+^, KLRG1^+^; *n* = 5 animals. (**D**) Frequency of CD127. MFI of STAT5 phosphorylation; *n* = 5 animals. (**E**) Histogram and MFI of CD127 in; *n* = 5 animals. (**F**) Frequency of EdU^+^ cells; *n* = 6 and annexin V^+^ cells; *n* = 4 animals. (**G**) Histograms of Helios and Neuropilin-1. (**H**) Thymocyte transplant assay. Counts of Foxp3^+^ cells 8 weeks after transplantation; *n* = 5 animals. Data are shown as mean ± SD; graphs represent data from at least 3 independent experiments. Statistics performed with 1-way ANOVA with Tukey’s multiple comparisons test at 95% CI; *P < 0.05, **P < 0.01, ***P < 0.001, ****P < 0.0001.

To evaluate the tissue-specific functions of BM Tregs (marrow Tregs), we compared their distribution to Tregs in other tissues: spleen and inguinal lymph node (LN). Consistent with the published literature, we found that the marrow was enriched in CD3^+^CD4^+^Foxp3^+^ cells. Notably, the frequency of Tregs is approximately 3 times greater in the marrow than other lymphoid organs (5, 13, 15, 35). Marrow Tregs compose ~40% of total CD3^+^CD4^+^ cells (Figure 1, A and B) and about 0.005%–0.01% of total BM cells, suggesting that they have important functions in this site.

To further profile marrow Tregs, we examined the expression of informative surface receptors (36) (Figure 1C and Supplemental Figure 1A; supplemental material available online with this article; https://doi.org/10.1172/jci.insight.135681DS1). We observed minimal differences in activation markers: CD103, CTLA-4, CD69, CD73, with the exception of increased PD-1. Though notable differences included increased levels of killer cell lectin-like receptor subfamily G, member 1 (KLRG-1), as well as decreased expression of CD25 (IL-2Rα) and ICOS (inducible costimulator) (Figure 1, Supplemental Figure 1A). Of note, KLRG1 is a marker of terminal differentiation and tissue specialization, suggesting that marrow Tregs represent a mature effector population (37–39). Intriguingly, we also observed high expression of CD127/IL-7Rα (Figure 1, D and E). Because the functions of Tregs are considered to be independent of IL-7, and Tregs are characterized by low CD127 expression (40, 41), we sought to determine if increased CD127 expression denoted differences in reactivity and signal transduction. Following IL-7 stimulation, we observed an increase in phospho-STAT5, indicating that marrow Tregs are indeed responsive to this cytokine. Our results also demonstrate that CD127 is an intrinsic characteristic of marrow Tregs and that IL-7 signaling may represent an adaptive mechanism in a context where IL-7 is the prevalent cytokine.

Our analysis also revealed differences in proliferation and apoptosis. Marrow Tregs had similar rates of proliferation and cell death as Tregs in the spleen (Figure 1F), but less than those in the LN. Similar to other tissue Treg populations (adipose, lung, and intestine), marrow Tregs are highly expressed the IL-33 receptor ST2 (Supplemental Figure 1B). Marrow Tregs also highly expressed Helios and Neuropilin-1, indicating thymic origin (Figure 1G). To validate this, we transplanted CD3^+^ DN (CD4^–^CD8^–^) thymocytes from Foxp3^+^ (CD45.1) mice and assessed Treg development in recipient tissues (CD45.2, non-GFP hosts) 8 weeks after transplantation. Our analysis revealed that a significant portion of Tregs in the marrow, as well as the spleen, were derived from transplanted thymocytes. Foxp3^+^ cells were detected in the LN but at significantly reduced proportions (Figure 1H). In conclusion, we ascertain that marrow Tregs harbor markers of tissue specialization and differ from those in the periphery with regard to their phenotype and signaling capacity.

### Tregs in the BM preferentially localize back to this site.

**Figure 2:**
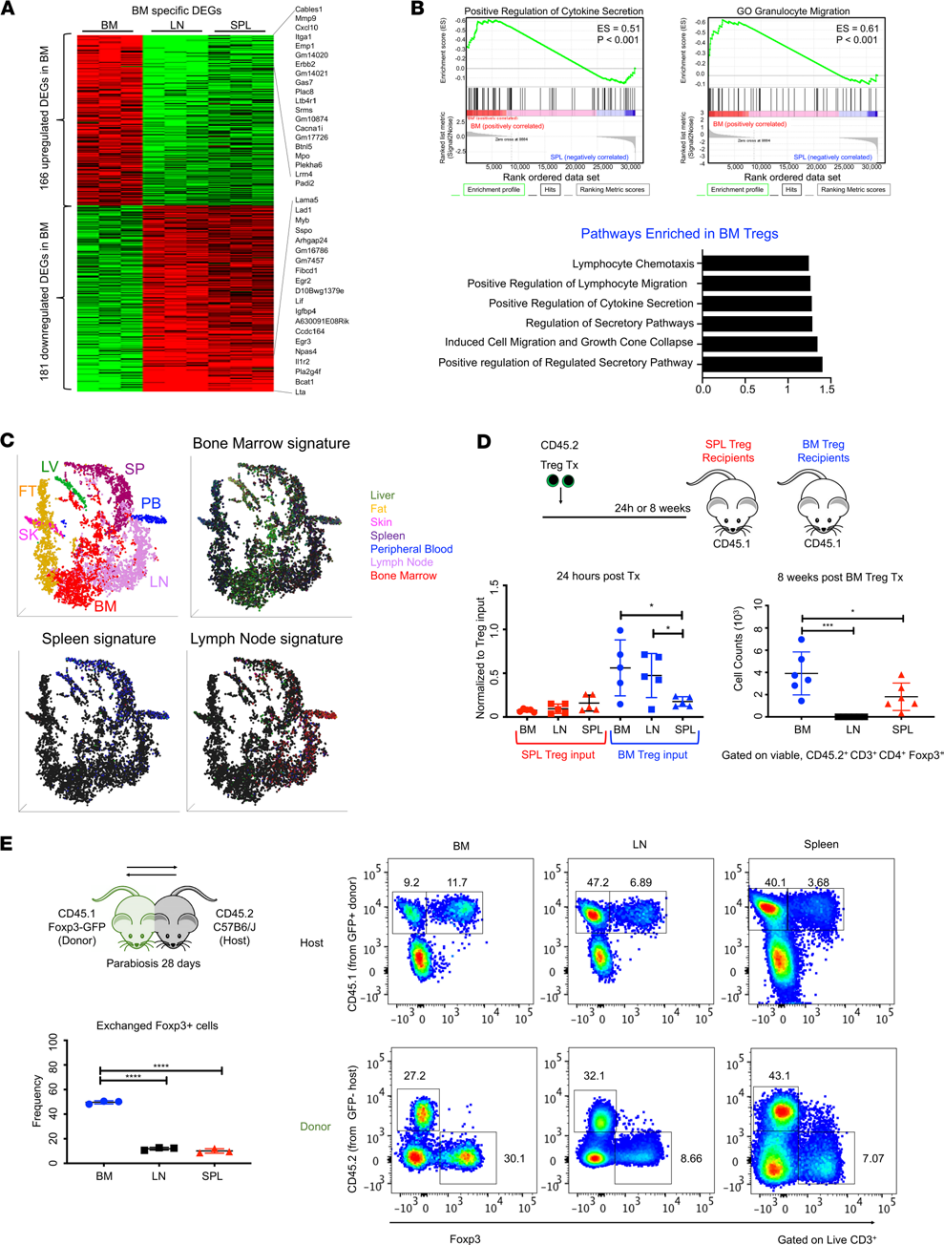
Marrow Tregs localize to their site of origin. (**A**) Heatmap showing DEGs in Foxp3^+^ cells; *n* = 3 replicates per tissue. DEG in marrow Foxp3^+^ cells. Genes with FC < 1/2 or > 2, and adjusted *P* < 0.05 are shown. (**B**) GSEA plot of marrow Foxp3^+^ cells (top) and pathway analysis (bottom); *n* = 3 replicates per sample. (**C**) An overlay of RNA-seq DEGs into t-SNE plots highlighting Treg-expressing signatures for bone marrow, spleen, and lymph node. (**D**) Homing measure 24 hours (left) and 8 weeks after transplantation (right). Data expressed as fold-change, normalized to input (24 hours); *n* = 5 or counts (8 weeks); *n* = 6 recipients. (**E**) Frequency of exchanged Foxp3^+^ cells (left). Plots of exchanged CD3^+^ populations (right); *n* = 3 parabionts. Data are shown as mean ± SD; graphs represent data from at least 3 independent experiments (**A**–**D**); 2 independent experiments (**E**). Statistics performed with 2-way ANOVA (**D**), 1-way ANOVA (**D** and **E**) with Tukey’s multiple comparisons test at 95% CI; *P < 0.05, ***P < 0.001, ****P < 0.0001.

To dissect transcriptional differences, we performed RNA sequencing (RNA-seq)analysis on Tregs isolated from the BM, LN, and spleen (GSE138095). We observed that marrow Tregs possessed a distinct transcriptome and were enriched for signatures related to homing, migration, secreted factors, and activation. We identified 347 differentially expressed genes (181 downregulated DEGs, 166 upregulated DEGs) in marrow Tregs. GSEA indicated an enrichment for genes associated with chemotaxis and enhanced cytokine secretion. Of note, the upper majority of differentially expressed genes were those encoding chemokine and cytokine receptors (Figure 2, A and B, and Supplemental Figure 2A). To compare marrow Tregs with Tregs in other tissues, we overlaid our bulk transcriptomic data with recently published single-cell RNA-seq (scRNA-seq) data sets (42). Pathways analysis and DEG signatures neatly distinguished marrow Tregs from other subsets according to their tissue-specific gene expression. Furthermore, unsupervised transcript clustering and t-distributed stochastic neighbor embedding (t-SNE) visualization revealed marrow Tregs to have a distinct transcriptome. Marrow Tregs converged to some degree with Tregs in visceral adipose tissue (VAT), as well as LN and peripheral blood — a finding that concurs with previous studies (9, 11, 43) and suggests that marrow Tregs might exchange with the circulating pool (Figure 2C and Supplemental Figure 2, B and C). We further analyzed the expression of CXCR4 and CD150 using transcriptomic data in combination with single-cell data sets of tissue Tregs. No striking transcriptional differences were detected, and we observed that CD150 and CXCR4 are expressed by Tregs in all tissues and not exclusive to marrow Tregs, contrary to other studies (Supplemental Figure 2, B and C). By surface analysis, we found equivalent CXCR4 expression across Treg subpopulations but did note an increase in CD150 in marrow Tregs, as has been reported (15, 43).

Across multiple tissues, the division of labor among Treg populations is maintained through synchronized homing patterns, which position Tregs at sites where regulation is required (11, 12). Thus, we evaluated the expression of various chemokine and homing receptors in marrow Tregs with respect to those from spleen and LN. We observed decreased expression of CD62L (L selectin), important for egress from lymphoid tissues and a marker for T cell development (44). We also noted increased expression of CD49b (integrin α_2_) relative to splenic cells, which delineates Treg specialization and correlates with enhanced effector function and migratory capacity (45) (Supplemental Figure 2C). Next, we tested if there was selective recruitment of Tregs to the BM using functional homing assays, as have been used to study HSCs (46). We transplanted Tregs isolated from different tissues and compared Treg migration with different lymphoid organs. Foxp3^GFP^ cells were injected into congenic nonirradiated mice, and homing to recipient tissues was assessed 24 hours after transplant. Remarkably, marrow Tregs had an enhanced ability to return to the BM — a characteristic resonant with stem cell function. Recipient marrows contained significantly higher numbers of marrow-isolated Tregs, implying that unique signaling cues concentrate Tregs back to this microenvironment. Interestingly, splenic Tregs did not preferentially home well to any tissue. We also assessed retention at extended time points and found that Tregs persisted in the marrow 8 weeks after transplant (Figure 2D). This demonstrates that marrow Tregs possess a distinctive ability to return to and persist in their tissue of origin. To assess the kinetics of Treg circulation, we generated parabiotic pairs of Foxp3^GFP^ mice (CD45.1) and non-GFP partners (CD45.2). Parabiosis generates a shared circulation system and is a well-validated method for studying T cell origin and trafficking (47, 48). After 28 days, parabionts and nonconnected controls were sacrificed, and cellular exchange was examined. Analysis revealed that marrow Tregs were highly migratory nonsessile cells (Figure 2E). At the tissue level, the marrow had lower cellular exchange than the spleen and LN (Supplemental Figure 2D). We also observed significantly decreased CD4^+^ chimerism (~25.6% exchange), whereas CD8^+^ exchange (~50.0%) was consistent across tissues. Although CD4^+^ T cell chimerism was decreased in the marrow, Treg exchange (~20.4%) was much higher than the LN (~5.5%) and spleen (~5.4%). Marrow Tregs were exchanged significantly more than other T cells (Foxp3^–^ CD4^+^ cells and CD8^+^ cells) (Supplemental Figure 2, E and F). This demonstrates increased affinity for the marrow environment and suggests that there may be targeted recruitment of marrow Tregs to this tissue. Collectively, our parabiotic analysis indicates that marrow Tregs are highly specific to the marrow and suggests that these cells may not need spleen or LN for instruction.

### Treg depletion does not result in BM T cell activation or inflammation.

**Figure 3:**
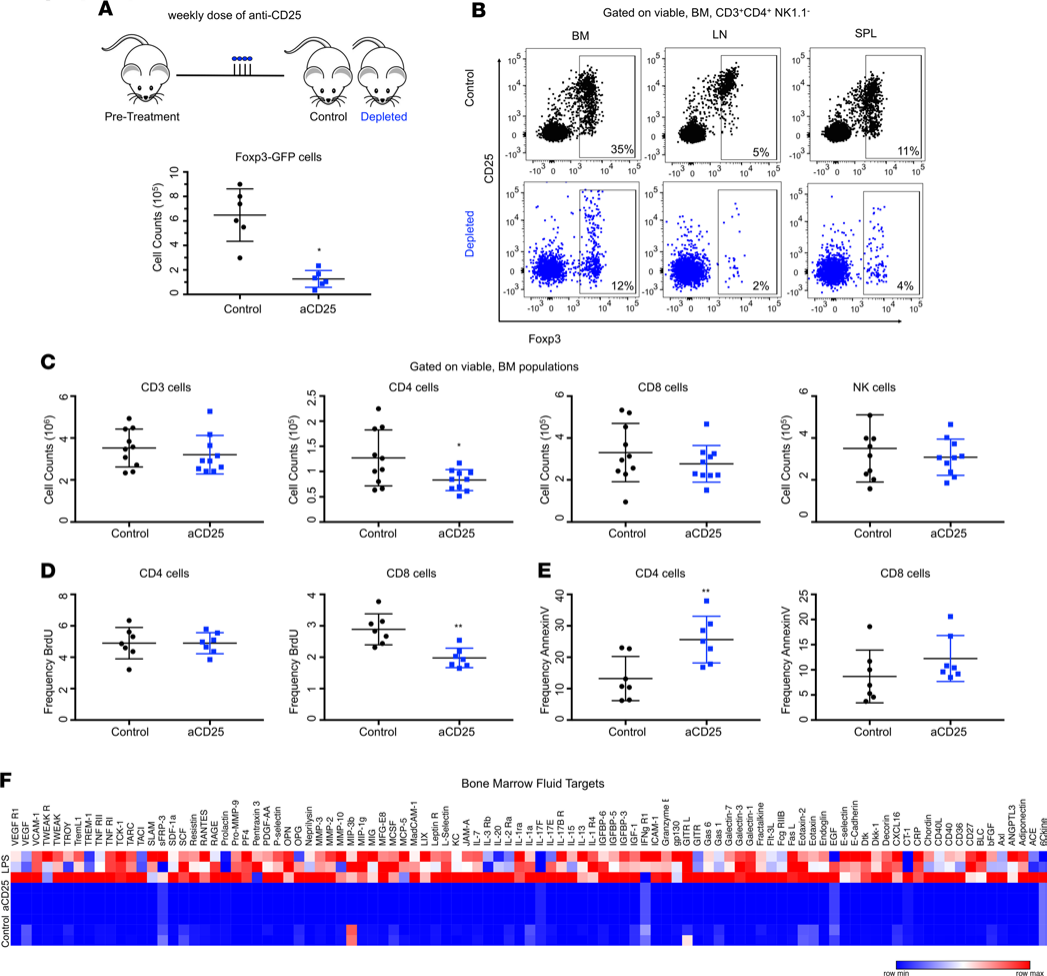
Treg depletion does not result in inflammation. (**A**) Counts of Foxp3^+^ cells; *n* = 6 animals per group. (**B**) Plot of Foxp3^+^ cells following anti-CD25. (**C**) Counts of marrow CD3^+^, CD4^+^, NK1.1^+^, and CD8^+^ cells; *n* = 10 animals per group. (**D**) Frequency of BrdU^+^ CD4^+^ and CD8^+^ cells; *n* = 7 animals per group. (**E**) Frequency of annexin V^+^ CD4^+^ and CD8^+^ cells; *n* = 7 animals per group. (**F**) Heatmap of top differentially expressed cytokines in marrow fluid; *n* = 3 replicates per condition. Differentially expressed cytokines defined with fold change (FC) < 0.5 or > 0.5 and adjusted *P* < 0.05. Data are shown as mean ± SD; graphs represent data from at least 3 independent experiments. Statistics performed with unpaired 2-tailed Student *t* test; **P < 0.01.

To test the physiological relevance of marrow Tregs, we assessed how marrow populations responded to their manipulation. Tregs were depleted via administration of anti-CD25 antibody. Using a low-dose regimen, we were able to specifically target Tregs, avoid depletion of other T cells, and circumvent the confounds of autoimmunity. anti-CD25–mediated Treg depletion led to a significant (<50%) reduction in Foxp3^+^ cells (Figure 3, A and B), as has been previously reported (49–51). Following depletion, we found no changes to tissue cellularity (Supplemental Figure 3A) and no significant expansion or increased proliferation of Foxp3^–^ CD4^+^, CD8^+^, or NK cells in the marrow, demonstrating that the primary function of marrow Tregs is not immunosuppression (Figure 3, C and D, and Supplemental Figure 3, B and C). This was surprising, given that the canonical function of Tregs is to limit effector T cell expansion and proliferation(52). While we observed a reduction of CD4^+^ cells numbers and increased CD4^+^ apoptosis, this can be specifically ascribed to the loss of CD4^+^ Tregs that undergo cell death as a consequence of anti-CD25 (Figure 3E and Supplemental Figure 3C). Overall, we observed no indication of overt inflammation among other mature populations in spleen or marrow, with the exception of a slight myeloid expansion (Supplemental Figure 3, D and E). Finally, to assess local and systemic effects of anti-CD25, we evaluated over 200 chemokine and cytokine targets in the serum and BM fluid of Treg-depleted mice. We detected a negligible increase of inflammatory markers (including IL-1, IL-17, IL-22, TNF-α, and IFN-γ) in the serum (systemic) or the BM fluid (local), with most targets relatively unchanged (Figure 3F and Supplemental Figure 3F). As a reference, we compared anti-CD25 depletion with a low-dose TLR mimic LPS, which produced < 100-fold change in expression. Only Artemis and IL-2Rα had < 2-fold change at 2.13- and 2.96-fold changes, respectively. Thus, we determined that there was no inflammation using this treatment. Altogether, this suggests that inhibition of T cell activation and cytokine-mediated inflammation is not the principal function of marrow Tregs.

### BM Tregs are required for stromal cell maintenance.

**Figure 4:**
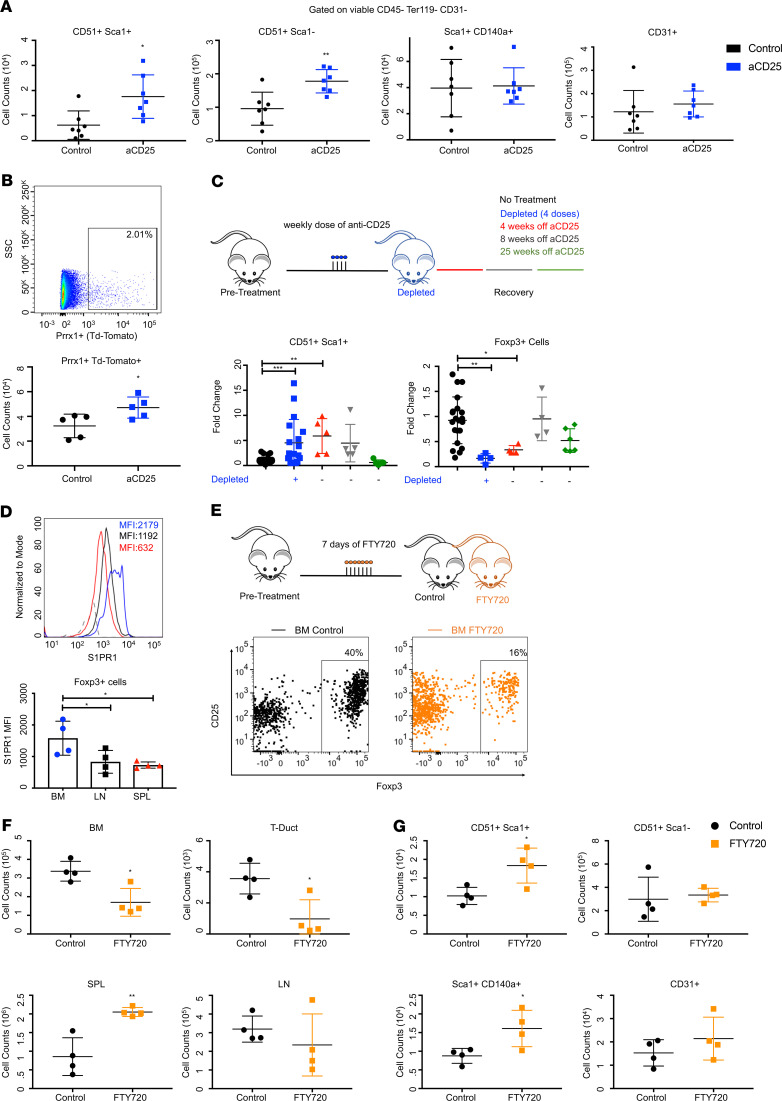
Tregs maintain stromal cell. (**A**) Counts of stromal populations following anti-CD25; *n* = 7 animals. (**B**) Plot and counts of TdTomato^+^ stromal cells; *n* = 5 animals. (**C**) Recovery after anti-CD25. Quantification of MSCs and Foxp3^+^ cells at 4, 8, and 25 weeks after anti-CD25. Data expressed as fold-change relative to untreated mice. (**D**) Histogram and quantification of S1PR1 MFI in Foxp3^+^ cells; *n* = 4 animals. (**E**) Representative plot of Foxp3^+^ cells after FTY720. (**F**) Counts of Foxp3^+^ cells following FTY720; *n* = 4 animals per group. (**G**) Counts of stromal populations following FTY720; *n* = 4 animals. Data are shown as mean ± SD; graphs represent data from at least 3 independent experiments. Statistics performed with unpaired 2-tailed Student *t* test (**A** and **B**, **F**–**G**) and 1-way ANOVA with Tukey’ multiple comparisons test at 95% CI (**C** and **D**); *P < 0.05, **P < 0.01, ***P < 0.001, ****P < 0.0001.

Given the crosstalk that exists between Tregs and their local tissue environments (4), we hypothesized that Tregs might regulate the function of nonhematopoietic cells within the marrow. Indeed, we observed an expansion and increased proliferation of various stromal populations following Treg depletion, including CD31^–^CD51^+^Sca1^+^ phenotypic MSCs and CD31^–^CD51^+^Sca1^–^ (bone precursors). A similar but not statistically significant trend was observed for Sca1^+^CD140α^+^ (PDGF-Rα) cells, and no significant changes were noted for endothelial cells (CD31^+^) (Figure 4A and Supplemental Figure 4A) (53–55). To validate this, we used a stromal-associated strain, Prrx1-Cre crossed to a ROSA/tdTomato reporter, in which tdTOM^+^ labeling corresponds to stroma. Following anti-CD25, the numbers of tdTOM^+^ cells were significantly increased, corroborating our initial findings (Figure 4B).

We then tested if the stromal phenotypes seen after Treg depletion could be rescued. We tracked the recovery of hematopoietic and stromal populations after stopping anti-CD25 treatment. We evaluated the stabilization of populations to baseline conditions (no treatment) during the course of short-term (4–8 weeks after discontinuing anti-CD25) and long-term (LT) (25 weeks) recovery. We observed that the normalization of the stromal compartment occurred proportionate to the restoration of Tregs (Figure 4C). This suggests that BM stromal cells are finely tuned to the presence of Tregs.

Our in vivo data suggest a meaningful role for Tregs with regard to stromal regulation. To validate this, we looked for methods that would specifically target the marrow Treg pool. One marker, sphingosine-1-phosphate receptor 1 (S1PR1), was highly expressed on marrow Tregs (Figure 4D). Since others have shown that both T cells and stem cells use gradients S1P to home back to the BM (56, 57), we investigated if the S1P/S1PR1 axis was a potential mechanism for Treg trafficking. Mice were treated with S1P receptor modulator phospho-fingolimod (FTY720), which interferes with S1PR signaling and abrogates the S1P/S1P1-dependent egress of lymphocytes from lymphoid tissues. Because S1P trafficking is highly tuned to the surface expression of different S1P receptors (S1PR1–5), the time-course and dose of FTY720 was chosen to limit off-target effects to other S1PR1-expressing cells, including lymphocytes and HSCs. Targeting S1PR1-mediated recirculation allowed us to prevent the influx of Tregs to the marrow and assess the cellular response (57). After 7 days of FTY720, we observed a significant decrease of Tregs in the marrow, suggesting that S1P/S1P1 gradients are involved in marrow Treg trafficking (Figure 4, E and F, and Supplemental Figure 4B). We specifically noted an accumulation of Tregs in the spleen, consistent with other reports (58). Importantly, this treatment did not alter the Treg numbers in the other peripheral tissues, allowing for more precise manipulation of marrow Tregs. With FTY720, we observed an expansion and increased proliferation of PDGFRα^+^ cells and MSCs (Figure 4G), similar to anti-CD25. No significant changes were observed for endothelial cells or bone progenitors. This substantiates our observations that stromal populations acutely respond to disruptions in the Treg pool.

### BM Tregs enhance niche-dependent support of HSCs.

**Figure 5:**
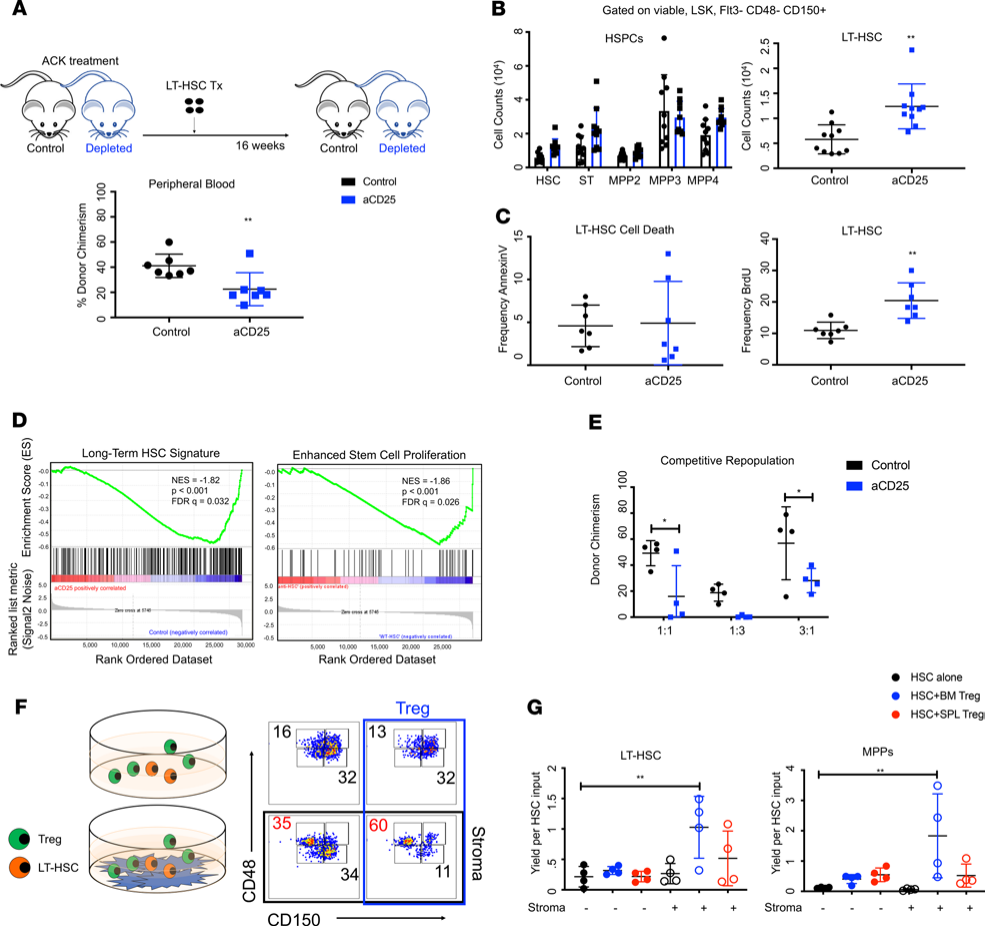
Marrow Tregs enhance HSC-supportive activity of stromal cells. (**A**) Clearance via ACK2 treatment and peripheral blood chimerism; *n* = 10 animals. (**B**) Counts of HSPCs following anti-CD25; *n* = 10 animals. (**C**) Frequency of annexin V^+^ and BrdU^+^ LT-HSC following anti-CD25; *n* = 8 animals. (**D**) GSEA plot for anti-CD25 LT-HSCs; *n* = 4 replicates. (**E**) Competitive repopulation assay and peripheral blood chimerism; *n* = 4 recipients. (**F**) Coculture experiment and representative flow plot of HSPC, Tregs, and stromal cells. (**G**) Quantification of HSCs and MPPs. Data expressed as fold change and normalized to input of HSCs; *n* = 4 wells per condition. Data are shown as mean ± SD; graphs represent data from at least 3 independent experiments. Statistics performed with unpaired 2-tailed Student *t* test (**A**–**E**) and 1-way ANOVA with Tukey’ multiple comparisons test at 95% CI of (**G**) *P < 0.05, **P < 0.01.

Stromal cells regulate important hematopoietic stem and progenitor cells (HSPCs), properties including self-renewal, differentiation, and quiescence via release of soluble factors and cell-cell interactions (20). The changes to the stromal compartments led us to hypothesize that Tregs may regulate important stromal cell functions, including HSC support. We focused on HSC support because blood stem cell regulation is a defining feature of the niche with immense clinical implications (59–62). To test the effect of Tregs on the HSC-stromal relationship, we assayed the ability of the Treg-depleted niche to support HSCs using nongenotoxic conditioning and transplantation. In order to maintain the existing niche architecture and circumvent the need for irradiation, recipients were cleared with ACK2, an antibody clone blocking c-Kit function (63). LT-HSCs were then transplanted into either WT or Treg-depleted mice (anti-CD25). We observed appreciably reduced peripheral blood engraftment at 16 weeks after transplant in the Treg-depleted mice (Figure 5A). These findings indicate that the capacity of the stromal niche to support LT-HSCs is impaired in the absence of Tregs. Importantly, it demonstrates that Tregs influence extrinsic, niche-dependent stem cell regulation.

Others have shown that HSPCs are sensitive to environmental changes and are influenced by stromal alterations (64–66). Consistent with this, we noted an expansion of HSPCs after anti-CD25–mediated Treg depletion (Figure 5B). Specifically, an increase in proliferation of LT-HSCs (Lin^−^Sca1^+^c-Kit^+^CD150^+^CD48^−^; LT-HSCs) was observed (Figure 5B) with no significant changes in cell death (Figure 5C) (67, 68). We also observed the presence of extramedullary hematopoiesis, as evidenced by the increase of LT-HSC to the spleen (Supplemental Figure 5C). To transcriptionally dissect this phenotype, we performed RNA-seq on LT-HSCs. We identified 179 upregulated DEGs from anti-CD25–treated mice. GSEA indicated a decrease in classical stem cell signatures, as well as increased proliferation (Figure 5D and Supplemental Figure 5A). We observed negligible alterations to more differentiated progenitor populations (Supplemental Figure 5B). Since HSCs are typically a quiescent population sensitive to environmental cues (69–71), the phenotypic and transcriptional profiles following Treg depletion suggested functional decline. To test this, we assayed the effects of Treg loss on intrinsic properties of LT-HSC. This was tested through competitive repopulation assays using different competitor donor ratios. At 16 weeks after transplant, we observed lower total peripheral blood chimerism in mice transplanted with cells from anti-CD25–treated mice (Figure 5E and Supplemental Figure 5D). This reduced repopulating capacity indicates a loss of functional potential. Together, these results indicate that stem cells from a Treg-depleted environment are phenotypically expanded and impaired, even when placed in a healthy microenvironment.

Because our in vivo treatments involved complex cellular interactions, we sought to dissect the effect of Tregs more directly through cocultures. To do this, we generated an ex vivo hematopoietic support assay in which HSC numbers act as a proxy for stem cell maintenance. In our assay, HSC numbers indicate stem cell survival and act as a surrogate measure of stromal cell function (HSC support). Although cocultures do not entirely replicate the complexity of the marrow microenvironment, they are among the better ex vivo assays available (72). We cultured HSCs in the presence or absence of stroma and assessed how Tregs altered this system (Figure 5F). In line with our in vivo observations, we found enhanced HSC support in conditions where stroma and Tregs were present. Marrow Tregs enhanced the ability of stromal cells to both maintain HSCs and promote maturation to phenotypic multipotent progenitors (Figure 5G). Importantly, this was exclusive to marrow Tregs, as splenic Tregs had no effect. Together with the transplant studies, this suggest that marrow Tregs enhance the capacity of stromal cells to support hematopoiesis and maintain stem cells.

### Treg-secreted IL-10 alters stromal cell proliferation, differentiation, and HSC support.

**Figure 6:**
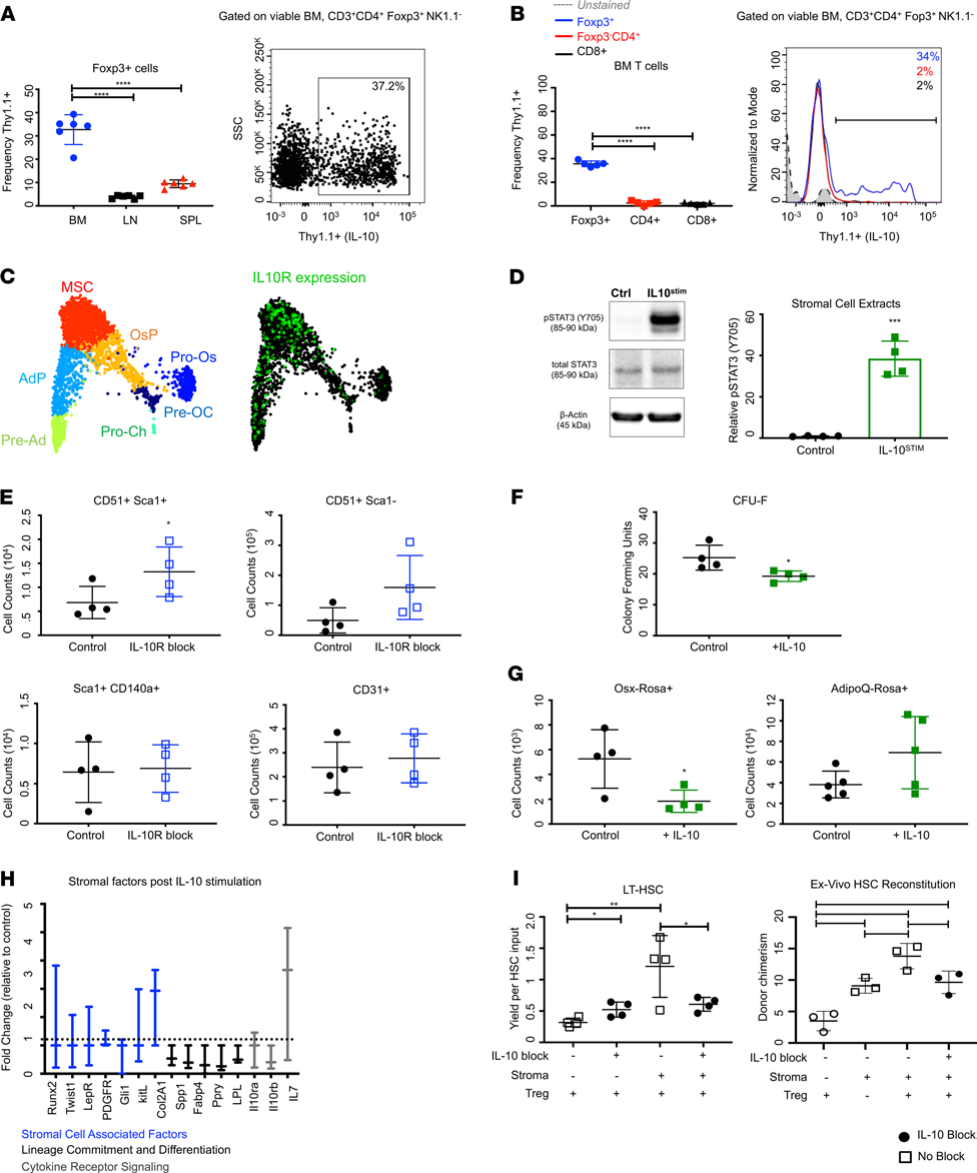
Treg IL-10 restricts stromal cell proliferation and differentiation. (**A**) Frequencies and plot of Thy1.1^+^Foxp3^+^ cells in bone marrow (right); *n* = 6 animals per group. (**B**) Frequencies and histogram of Thy1.1^+^ cells: CD4^+^Foxp3^+^(blue), CD3^+^CD4^+^Foxp3^–^(red), CD3^+^CD8^+^(black); *n* = 5 animals. (**C**) SPRING plots of stromal cells transcriptomes. Preadipocyte (pre-Ad), Adipocyte progenitor (AdP), Mesenchymal stromal cell (MSC), osteoblast/chondrocyte progenitor (OsP), preosteoblast/chondrocyte (Pre-OC), pro-osteoblast (Pro-Os), and prochondrocyte (Pro-Ch). Relative abundance of IL-10Ra (green). (**D**) Western blot of STAT3 phosphorylation (Tyr 705) and densitometric protein analysis; *n* = 4 biological replicates. (**E**) Counts of stromal populations in IL-10R^BLOCK^ mice, or blockade of IL-10R via injection of anti–mouse IL-10R; *n* = 4 animals per group. (**F**) CFU-Fs of stromal cells following 7 days of IL-10 stimulation; *n* = 4 wells per condition. (**G**) Counts of TdTomato^+^ cells from Osx-Cre and AdipoQ-Cre mice following 7 days of IL-10 stimulation; *n* = 4 wells per condition. (**H**) qPCR analysis of transcripts following 7 days of IL-10 stimulation. Genes presented as fold change (relative to control); *n* = 3 biological replicates; each point was done in triplicate (normalized to TBP1). (**I**) Coculture of LT-HSCs, Foxp3^+^ cells, and stromal cells with IL-10–blocking antibody for 96 hours; *n* = 4 wells per condition (left). Transplantation assay of ex vivo HSCs collected from individual coculture wells after 96 hours of exposure. Peripheral blood chimerism of donor cells; *n* = 3 recipients per group (right). Data are shown as mean ± SD (**A** and **B**, **E**–**G**, **I**) or mean ± SEM (**H**); graphs represent data from at least 3 independent experiments. Statistics performed with unpaired 2-tailed Student *t* test (**E**–**G**) and 1-way ANOVA with Tukey’ multiple comparisons test at 95.00% CI of diff (**A**, **B**, and **I**); *P < 0.05, **P < 0.01, ***P < 0.001, ****P < 0.0001.

A key cytokine associated with Treg function is IL-10. This pleiotropic antiinflammatory cytokine allows Tregs to temper immune responses at various tissue interfaces (73–75). We used a previously established IL-10 reporter system (in which IL-10 expression is linked to Thy1.1) to analyze IL-10 production in vivo (76, 77). We identified that marrow Tregs are high producers of IL-10 and generate significantly more IL-10 transcripts than other Tregs (Figure 6, A and B). We also uncovered that Tregs are a principal source of IL-10 in BM (Supplemental Figure 6A; see complete unedited blots in the supplemental material). To resolve potential targets of Treg-secreted IL-10, we analyzed scRNA-seq data sets for the expression of IL-10 receptor α (IL-10Rα) across mature, progenitor, and stromal BM populations (78, 79). Intriguingly, MSCs highly expressed the IL-10Rα. They also had the highest IL-10Rα expression across stromal populations (Figure 6C and Supplemental Figure 6B) (78). We detected no IL-10Rα expression in HSPCs. To probe this mechanism, we stimulated stroma with IL-10 and evaluated downstream signaling targets (Figure 6D). Indeed, IL-10 stimulation triggered robust STAT3 phosphorylation (40-fold increase in pSTAT3), indicating that stroma actively respond to IL-10 signaling.

To determine if the stromal response was brought on by disruptions to IL-10, we performed in vivo neutralization of IL-10 signaling by blocking the IL-10R (via anti–IL-10R). We observed that IL-10R blockade reproduced the stromal phenotypes seen with Treg depletion via anti-CD25 (Figure 4A and Supplemental Figure 4A) and Treg sequestration via FTY720 (Figure 4G). This resulted in expansion of MSCs and bone-forming progenitors (Figure 6E). No significant changes were observed for endothelial or PDGFRα^+^ cells. We observed no significant changes to mature myeloid or T cell populations, but we did recapitulate the expanded LT-HSC phenotype (Supplemental Figure 6, C and D). These results concur with our other transient drug treatments and indicate that IL-10 selectively and specifically regulates both stromal cells and HSCs.

The robust stromal response to IL-10 led us to hypothesize that Treg-secreted IL-10 may have a conditioning role in shaping the BM microenvironment. To assess its LT influence, we analyzed mice with selective KO of IL-10 in Foxp3-expressing cells (Foxp3-Cre × IL-10^fl/fl^). Intriguingly, lifelong genetic deletion of Treg-derived IL-10 resulted in a reduction of MSCs, bone-forming progenitors, and PDGFRA^+^ cells (Supplemental Figure 6E). Endothelial cells remained unchanged. This blunted phenotype suggests that Treg-secreted IL-10 is necessary for normal stromal development. We also observed an expansion of myeloid populations and no significant changes to T cells. In contrast to our transient treatments, no major changes were observed in HSPCs (Supplemental Figure 6, F and G). To further dissect the developmental influence of IL-10 on stromal cells, we generated Prrx1-Cre × IL-10R-α^fl/fl^ mice in which IL-10Rα was deleted from Prrx1-expressing stromal populations. With this additional lifelong genetic deletion model, we recapitulated the blunted stromal phenotype. We observed a significant reduction in stromal populations similar to Foxp3-Cre × IL-10^fl/fl^ mice (Supplemental Figure 6H). These findings highlight the differential effects between transient and prolonged IL-10 perturbations on stroma. Our observations suggest that acute perturbations of Tregs/IL-10, as seen with anti-CD25, IL-10R blockage, and FTY720, lead to increased stromal proliferation, consistent with a stress response (80). Conversely, lifelong loss of IL-10 signaling, as seen with genetic deletions, appears to hamper stromal cell maturation, resulting in decreased numbers. These findings suggest that Treg-derived IL-10 may have additional importance for shaping the developmental trajectories of stromal cells.

To gain a better understanding for the role of IL-10 on stromal differentiation and lineage commitment, we used the colony-forming unit (CFU) assay to test if IL-10 influenced the clonogenic potential of stromal progenitors. Stroma stimulated with IL-10 showed a ~10% reduction in CFU activity, indicating that IL-10 limits colony formation (Figure 6F). Additionally, we tested how IL-10 influenced differentiation into bone and fat lineages. We used 2 additional stromal-associated strains: Osx-Cre (early bone-forming cells) and AdipoQ-Cre (adipocyte cells) crossed to ROSA/tdTomato reporters (81, 82). We observed fewer tdTOM^+^ cells in Osx cultures, indicating that IL-10 alters bone lineage commitment. No consistent differences were observed regarding adipocyte differentiation (Figure 6G). We also evaluated the expression of stromal-associated factors upon IL-10 stimulation. Upon stimulation, we detected increased levels of stromal-associated transcripts *Runx2*, *LepR*, and *PDGFRa*, and *KITLG*, which have been associated with enhanced hematopoietic support (22, 83, 84). Conversely, transcripts associated with lineage commitment, including *Col2A1*, *Spp1*, *Ppry*, and *Fabp4,* were expressed at lower levels (33, 54, 85, 86). Interestingly, the receptor for IL-10, *IL-10ra*, was also upregulated, as was IL-7, implying that a feedback loop might exist between Tregs and stroma (Figure 6H). These findings demonstrate that IL-10 is an important mediator of stromal-secreted factors and suggest that IL-10 signals may serve as checkpoints for stromal precursors to advance cell lineage decisions.

Finally, we sought to determine if stromal cell function (specifically HSC maintenance) was directly affected by Treg-secreted IL-10. To test this, we evaluated support of HSC in the presence of Tregs using our coculture model, but this time, we introduced a neutralizing antibody to IL-10. We again tested phenotypic HSC maintenance ex vivo because it provides a short-term read-out of one of the most clinically relevant stromal cell functions. Notably, we observed that neutralization of IL-10 significantly abrogated the capacity of stromal cells to maintain phenotypic HSCs (even in the presence of Tregs). This indicates that IL-10 is a critical actor in Treg regulation of stromal cells (Figure 6I).

To validate the phenotypic HSC observations from the ex vivo cultures, we tested the functional capacity of HSCs after 96 hours of coculture with Tregs, stroma, and IL-10 neutralizing antibody. This was done by transplanting HSCs from individual wells and evaluating engraftment and LT lineage reconstitution (Figure 6I and Supplemental Figure 6I). Analysis of donor chimerism revealed that even short-term ex vivo exposure to marrow Tregs was sufficient to enhance HSC support and improve engraftment. Critically, the Treg effect on engraftment was abrogated in cohorts where IL-10 was neutralized. In the absence of other cellular actors, this suggests that Treg-derived IL-10 directly augments the ability of stromal cells to support HSCs. These findings expand on the conclusions from our coculture analysis as HSCs demonstrating that stem cells are not just phenotypically maintained, but also functionally improved by presence of marrow Tregs and stroma. We also propose that this ex vivo culture system is a useful model to functionally evaluate the effects of Treg IL-10 on stromal cell function in absence of other cellular variables.

In line with the previous profiling, we also observed decreased HSC maintenance using stroma derived from Foxp3-Cre×IL-10^fl/fl^ mice (Supplemental Figure 6J). This suggests that stroma with functional IL-10 signaling are more efficient at ex vivo HSC support. Together with the transplant studies, this suggest that marrow Tregs enhance the capacity of stromal cells to maintain stem cells via IL-10. The data further demonstrate that IL-10 has a dual role: it regulates the differentiation trajectory of MSCs and enhances their function with regard to secreted factors and stem cell maintenance.

## Discussion

Our study sheds light on the defining properties of marrow Tregs and outlines an uncovered role for Treg-secreted IL-10 in regulating stromal cell function and development. We found that Tregs are enriched within the marrow and are specialized for the maintenance of this tissue. Furthermore, transcriptional and functional characterization of marrow Tregs identified a unique profile of chemokine receptors, surface markers, and circulation patterns. Notably, depletion of Tregs showed no increases in T cell proliferation proinflammatory cytokines, suggesting that their role in the marrow is not immunosuppression. In contrast, stem cells and MSCs were severely altered by Treg depletion. Importantly, the effects of Treg depletion were replicated with different methods of manipulation, including Treg sequestration and blockade of IL-10 signaling, underscoring the importance of Treg-derived IL-10 as a key mediator of these phenotypes.

Our work provides a comprehensive evaluation of BM Tregs in relation to those in the periphery, as well as tissue-specific subsets. We conclude that BM Tregs are tissue specialized. Marrow Tregs express genes associated with activation and terminal differentiation, and they harbor a unique profile of chemokine receptors. Another distinguishing property of marrow Tregs was high expression of CD127. In the context of Treg biology, this could be an environmental adaptation to the low IL-2 levels in the marrow, and it is likely that Tregs rely on IL-7, provided by the surrounding stroma (87). Our observations are in line with studies showing colocalization of BM memory CD8^+^ T cells in the proximity of IL-7–producing stromal cells (88). In fact, the decrease in proliferation of CD8^+^ T cells we observed upon Treg depletion may itself be a result of stromal alterations. Given that the marrow serves a memory T cell niche, our CD127 phenotype supports the notion that Tregs in different environmental contexts can be sustained through IL-7 and can signal through CD25-independent mechanisms.

We have also identified a clinically relevant mechanism to disrupt Treg trafficking to the BM. We provide evidence that marrow Tregs depend on S1P gradients for migration and that disruption of this axis allows for sequestration of Tregs out of the marrow. More detailed investigations into S1P-mediated Treg trafficking are required to further develop this system for Treg manipulation. Remarkably, marrow Tregs are able to home back to the marrow, and they persist in this tissue in a similar fashion as HSCs (56). We also demonstrate that marrow Tregs are highly migratory and not tissue resident. This circulatory profile was corroborated by parabiosis. We propose that the homing capacity of marrow Tregs makes them an attractive candidate for targeted drug delivery. Importantly, it raises the possibility that stem cell therapy, GVHD, and transplantation could be enhanced via marrow Tregs because these cells equipped to provide site-specific regulation and can directly target mesenchymal components of the BM.

In other studies, localization of Tregs to the marrow was attributed to increased expression of CXCR4 (15). We evaluated CXCR4 expression at the protein, bulk transcript, and single cell levels and observed no differences. Because marrow Tregs are highly circulatory, a Foxp3-specific deletion of CXCR4 likely prevents Treg reentry to the marrow, resulting in diminished numbers, but also targets peripheral Tregs nonspecifically. Furthermore, manipulation of CXCR4 negatively alters Treg function, as CXCR4 antagonists have shown to inhibit Tregs’ suppressive activity (89). Given the multiple roles that CXCR4 has in cellular trafficking to the marrow, and T cell trafficking specifically, disruption of CXCR4 signaling does not represent a translationally feasible mechanism by which to target the marrow Treg pool.

Upon Treg depletion, a significant expansion of HSPCs and stromal cells was observed, as well as increased proliferation of HSCs. We propose that this is a direct consequence of the alterations imparted on the marrow microenvironment. Moreover, the absence of Tregs compromised the capacity of stromal cells to support HSCs both in vivo and in coculture. Using 3 different methods — Treg depletion, Treg sequestration, and IL-10R blockade — we have shown that loss of marrow Treg function has a critical effect on HSCs in a stromal cell–dependent manner. This indicates that marrow Tregs directly regulate the mesenchymal components responsible for maintaining HSCs, and our work supports the notion that Tregs act on HSCs both directing and indirectly via modulation of the stroma niche.

In the process of selecting a method for Treg depletion, we also evaluated a Foxp3–diptheria toxin receptor (Foxp3-DTR) model where Foxp3^+^ cells are ablated following DT administration (90). While this is a widely accepted immunological model, DT regimens produce unwanted side effects (91, 92). In Foxp3-DTR mice, we observed accelerated deterioration and rapid onset of autoimmunity, including increased cell death. Furthermore, multiple populations directly respond to inflammatory stimuli, and these represent off-target effects incompatible with our studies. Given the therapeutic potential of Tregs, we instead aimed to use models with translational applicability. Thus, a significant advantage of anti-CD25 administration is that the humanized anti-hCD25 antibody (daclizumab) has been clinically validated (51, 93).

Other studies have demonstrated that MSCs enhance the function of various immune cells — and of Tregs, specifically (94–101). However, few have addressed if the reverse also occurs. Similarly, while it is known that IL-10 is critical for immunosuppression (74, 75, 102), a role for IL-10 in hematopoiesis and in stromal cell conditioning was previously unexplored. Here, we identified a unique mechanism for Treg-derived IL-10 in shaping a tissue niche. We demonstrate that IL-10 influences stromal capacity to support HSCs, consistent with studies in other tissues, for example, of IL-10 acting on intestinal epithelial cells (103). While this does not obscure the potential for Tregs to regulate stromal cells via other cell-cell contact mechanisms or secreted factors, it offers a viable target for modulating the stromal environment. We observed changes to stroma when we manipulated IL-10 signaling in an acute manner (via loss of Tregs or blocking IL-10 signaling) and systemically using a lifelong elimination of Treg-secreted IL-10 (Foxp3-Cre × IL-10^fl/fl^) or IL-10 stromal cell signaling (Prrx1-Cre × IL-10R^fl/fl^) mice. We attribute the disparity in some phenotypes to the differences in treatment length (acute versus LT) and animal models: transient treatments versus genetic deletions (lifelong IL-10 perturbations). With the transient treatments of anti-CD25, IL-10R blockage, and FTY720, the loss of IL-10 signaling is temporary, resulting in increased proliferation and consistent with a stress response expansion (80). Conversely, we propose that IL-10 is critical for MSCs. Thus, having limited access to a major source of IL-10 from birth likely blunts stromal cell development, resulting in decreased numbers. Collectively, both observations support a model in which the stromal alterations are precipitated by the absence of IL-10–producing Tregs. Our transplantation and ex vivo assays demonstrate that Tregs render the BM niche more supportive and functional. Translationally, it may even be possible to improve strategies for ex vivo HSC expansion in culture by advancing our understanding of how immune cells influence the niche-dependent mechanisms that promote HSC maintenance. We propose Treg-derived IL-10 directly contributes to HSC support in a stromal cell–dependent manner. As Treg dysregulation has been implicated in numerous diseases, future studies into how IL-10 signaling is dysregulated in the context of inflammation or hematological malignancy represent promising areas of investigation.

Overall, this work sheds light on how mature immune cells actively regulate hematopoiesis via the local microenvironment. We have discovered a unique Treg–stromal cell interaction pathway, which reconciles previous investigations on marrow Tregs and expands our understanding of their tissue specialization. These studies provide a foundation for the development of therapies aimed to manipulate stromal populations and enhance their function. This has broad implications for the treatment of hematological malignancies, transplantation, and the emerging fields of Treg and mesenchymal cell therapy.

## Methods

### Anti-CD25.

A total of 0.5 mg/mouse of InVivoMAb anti-mouse CD25 (PC-61.5.3) or InVivoMAb anti–rat IgG1 isotype control (anti-horseradish peroxidase) was administered i.p. weekly for 4 weeks before mice were euthanized for analysis.

### Blockade of IL-10R.

A total of 100 μg InVivoMAb anti–mouse IL-10R (1B1.3a; Bio X Cell) or vehicle (PBS) was administered 4 times at 5-day intervals. Mice were euthanized for analysis 10 days after last injection.

### EdU.

A total of 1 mg/mouse of EdU in 200 μL PBS was administered i.p. 16 hours before being euthanized for analysis. Cells were analyzed 16 hours after injection, and EdU incorporation was assessed according to the manufacturer’s protocol.

### BrdU.

A total of 1 mg/mouse of BrdU in 200 μL PBS was administered i.p. 16 hours before being euthanized for analysis. Cells were analyzed 16 hours after injection, and BrdU incorporation was assessed according to the manufacturer’s protocol.

### Annexin V.

Analysis of apoptotic cells were performed using APC-conjugated annexin V according to the manufacturer’s protocol (BioLegend)

### STAT5.

Cells were isolated from of 6- to 8-week-old *Foxp3*^GFP^ mice and incubated for 15 minutes at 37°C with or without recombinant murine IL-7 or IL-10 (PeproTech). Cells were processed according to manufacturer’s protocol (BD Phosflow Perm Buffer III). Cells were then stained with primary anti-Stat5 (pY694) or isotype control (BD Biosciences).

### FTY720.

FTY720 (reconstituted to 1 mg/mL in 1% DMSO and PBS). A total of 1 mg/kg FTY720 or PBS was administered i.p. weekly for 7 days before being euthanized for analysis.

### Bulk RNA-seq.

Raw files for RNA-seq for BM, spleen, and LN Tregs and for control and anti-CD25 LT-HSCs can be accessed with the GEO accession no. GSE138095.

### Tregs from lymphoid tissues.

Live CD4^+^CD8α^−^Foxp3^+^NK-1.1^−^ Tregs were sorted from these tissues (BM, inguinal LN, and spleen) of 6- to 8-week-old *Foxp3*^GFP^ mice and resuspended in Buffer RLT. RNA was extracted using RNeasy micro kit (QIAGEN), with 3 biological replicates per tissue.

### HSCs.

Live Lin^−^Sca-1^+^c-Kit^+^Flt3^–^CD150^+^CD48^−^ LT-HSCs were sorted from 10- to 12-week-old B6 mice treated with anti-CD25 or InVivoMAb anti–rat 34 IgG1 isotype control (anti-horseradish peroxidase) and resuspended in Buffer RLT (complete catalog information, including manufacturer and clones, for all antibodies is listed in Supplemental Table 1). RNA was extracted using RNeasy micro kit (QIAGEN), with 4 biological replicates per treatment condition.

### Library amplification and analysis.

Libraries were prepared using the TruSeq RNA Library Preparation Kit v2 (catalog RS-122–2001, Ilina) according to the manufacturer’s protocol. Sequencing was performed on the Illumina HiSeq2500. Reads of RNA-seq data were mapped to mouse mm10 genome using STAR aligner. Mapped read numbers of each gene were used to identify gene expression by feature Counts. Gene expression counts were further normalized among samples based on the total number of all mapped reads and were subsequently log_2_ transformed. Normalized data were further employed to identify differentially expressed genes with cutoff values of 2 for the average fold change between the comparing 2 groups and 0.05 for the *P* value of 2-tailed *t* test cross the replicated samples. DAVID (https://david.ncifcrf.gov/) was used for GO pathway analysis of differentially expressed genes. Hierarchical clustering with average linkage was performed for the normalized expression data across different tissues, and subsequently, the heatmaps were generated from the clustering results. Gene set enrichment analysis (GSEA) was performed based on the normalized data using GSEA v2.0 tool (http://www.broad.mit.edu/gsea/).

### Stromal cell isolation.

Nonhematopoietic stromal cells were processed as follows: residual bone fragments were crushed and digested in PBS and collagenase/dispase (1 mg/mL, catalog 11097113001, MilliporeSigma), for 45 minutes at 37°C, and then combined with the single cell BM suspension.

### Treg homing assay.

FAC-sorted Foxp3^+^ cells were isolated from BM or spleen of CD45.2^+^ mice were transplanted retro-orbitally into CD45.1 non-GFP recipients (20,000 cells per mouse). Recipient groups received either BM or spleen Foxp3^+^ cells. Mice were euthanized 24 hours after transplantation, and homing/distribution of Foxp3^+^ cells to BM, LN, and spleen of CD45.1 non-GFP recipients was assessed.

### Thymocyte transplant assay.

FAC-sorted CD3^+^ DN (CD4^–^CD8^–^Foxp3^–^) thymocytes from Foxp3^+^(CD45.1) mice were transplanted retro-orbitally into (CD45.2) non-GFP hosts (50,000 cells per mouse). Mice were euthanized 8 weeks after transplantation, and generation of Foxp3^+^ cells in BM, LN, and spleen of CD45.2 non-GFP recipients was assessed.

### ACK2 administration.

Antibody conditioning and transplantation was done in 2 rounds. A total of 500 μg of purified anti-mouse CD117 (c-Kit) antibody (clone ACK2; Supplemental Table 1) was administered retro-orbitally into CD45.2 mice. Seven days after ACK2, 500 sorted CD45.1^+^ HSCs (Lin^–^c-Kit^+^Sca1^+^CD150^+^CD48^–^) were transplanted. Mice were allowed to recover for 7 days. After recovery, they were reconditioned with another 500 μg of ACK2 and transplanted an additional 500 CD45.1^+^ HSCs 7 days later.

### Quantitative PCR.

Passage 1 stroma were cultured in the presence or absence of IL-10 (10 ng/mL) for 7 days, RNA was extracted using RNeasy micro kit (QIAGEN), and cDNA was synthesized using the Superscript III First-Strand Kit (Invitrogen). Quantitative PCR (qPCR) was performed in triplicate using Invitrogen SuperScript IV Reverse Transcriptase kit. Power SYBR Green Master Mix gene-expression assays were performed for murine Runx2, Twist1, LepR, PDGFR, Gli1, kitL, Angpt2, IL-10Rα, IL-1Rβ, col2A1, Spp1, Fabp4, Ppry, LPL, and IL-7 and were normalized to the level of TBP1 mRNA.

### Flow cytometry and cell sorting.

Single cell suspensions from BM, inguinal LN, thoracic duct, spleen, and peripheral blood were analyzed using a BD Fortessa X-20 flow cytometer or BD LSRII (BD Biosciences) sorted using a FACSAria (BD Biosciences). Diva software (BD Biosciences) and FlowJo (Tree Star Inc.) was used for data acquisition and analysis, respectively. Surface markers include KLRG1, ICOS, CD25, CD103, CD44, CD62L, CD49b, CD127, CTLA-4, PD-1, CD73, CD69, CXCR4, CD150, S1PR1, and ST2.

Mature populations were defined as follows: CD4^+^ T cell (CD3^+^CD4^+^NK1.1^–^), CD8^+^ T cell (CD3^+^CD8^+^NK1.1^–^) Treg (CD3^+^CD4^+^Foxp3^+^NK1.1^–^), NK T cells (NKT; CD3^+^NK1.1^+^), neutrophils (Gr-1^+^CD115^–^), macrophages (Gr-1^–^F4/80^+^), B cells (CD19^+^B220^+^), and immature-B cells (CD43^+^CD19^+^B220^+^).

HSPCs were defined as follows: granulocyte-macrophage progenitors (GMP; Lin^−^Sca-1^−^c-Kit^+^CD34^+^FcγR^+^), megakaryocyte-erythroid progenitors (MEP; Lin^−^Sca-1^−^c-Kit^+^CD34^–^FcγR^−^), LT-HSC (Lin^−^Sca-1^+^c-Kit^+^Flt3^–^CD150^+^CD48^−^), short-term HSCs (ST-HSC; Lin^−^Sca-1^+^c-Kit^+^Flt3^–^CD150^−^CD48^−^), and multipotent progenitors (MPP; Lin^−^Sca-1^+^c-Kit^+^Flt3^–^CD48^+^).

Nonhematopoietic stromal cells were defined as follows: nonhematopoietic, nonendothelial cells (CD45^–^Ter119^–^CD31^–^), endothelial cells (CD45^–^Ter119^–^CD31^+^), MSCs (CD45^–^Ter119^–^CD31^–^CD51^+^Sca-1^+^), osteoblasts (CD45^–^Ter119^–^CD31^–^CD51^+^Sca-1^–^), PDGFRA^+^ cells (CD45^–^Ter119^–^CD31^–^CD140α^+^Sca-1^+^).

Donor cell engraftment and lineage contribution was determined via analysis of the peripheral blood using anti-CD45.1 and -CD45.2 antibodies and lineage markers CD3e for T cell lineage, B220 for B cell lineage, and CD11b for myeloid lineage.

For complete antibody information, please refer to Supplemental Table 1.

### Stromal cell culture.

All cultures with stromal cells were carried out with murine stromal cells harvested from femurs and tibias and cultured through one passage (P1) and supplemented with Gibco MEMα, nucleosides, No Phenol Red Media supplemented with 100 U/mL penicillin-streptocycin, 100 U/mL antibiotic antimycotic solution (Lonza BioWhittaker Antibiotics: Penicillin-Streptomycin Mixtures) and 20% FBS (Denville). Media and cytokines were replaced every 3 days.

### Stromal Treg and HSC coculture.

LT-HSC (Lin^−^Sca-1^+^c-Kit^+^Flt3^–^CD150^+^CD48^−^) were cultured with Tregs sorted from BM or spleen (CD3^+^CD4^+^Foxp3^+^) at a 2:1 ratio with or without stromal cells (200 sorted CD45^–^/Ter119^–^/CD31^–^ cells) in Lonza BioWhittaker X-VIVO 15 Hematopoietic Serum-Free Culture Media supplemented with 100 U/mL penicillin-streptocycin, 100 U/mL Antibiotic Antimycotic Solution, 20 ng/mL murine SCF, 50 ng/mL Flt3, 10 ng/mL IL-3, 50 ng/mL IL-2, and 50 ng/mL IL-7. Cultures were supplemented with 10 ng/mL of IL-10 neutralizing antibody at a neutralization Dose (ND50) of 0.015 μg/mL, as indicated. Cultures were carried out for 96 hours in a 96-well flat-bottom plate.

### MSC CFU-fibroblast (CFU-F) assay.

A total of 10,000 stromal cells was cultured in a 24-well plate for 7 days supplemented with Gibco MEMα, Nucleosides, No Phenol Red Media supplemented with 100 U/mL penicillin-streptocycin, 100 U/mL Antibiotic Antimycotic Solution, and 20% FBS in the presence or absence of IL-10 (10 ng/mL). Media and cytokines were replaced every 3 days. Cells were rinsed with PBS, fixed with methanol, and stained with Wright-Giemsa Stain (MilliporeSigma). Cell clusters with more than 20 cells were counted as a colony.

### Western blot and protein quantification.

The cells were lysed using 50 μL of RIPA lysis buffer per 1 million cells. SDS-PAGE gels (10%) were loaded with 30 μg of protein for separation and then transferred to a nitrocellulose membrane using a Trans-Blot Turbo System (Bio-Rad). The membrane was blocked in TBST with 5% nonfat skimmed milk for 1 hour at room temperature. Blots were probed for pSTAT3-Y705 (D3A7 XP, Cell Signaling Technology) and reprobed for tSTAT3 (F-2, Santa Cruz Biotechnology Inc.). The images were taken using a GBox imager. Densitometry analysis of the blots was carried using ImageJ (NIH) normalized to the housekeeping gene B-Actin (C4, Santa Cruz Biotechnology Inc.).

### Competitive repopulation.

CD45.2 BM cells from control or anti-CD25 treated mice (competitors) were mixed with WT BM cells (donors), and a total of 5 × 10^6^ cells was injected into lethally irradiated CD45.1^+^ recipients. Donor competitor/donor ratios of 1:1, 1:3, and 3:1 were used.

### Ex vivo HSC transplantation assay.

CD45.2^+^ HSCs were collected from individual coculture wells after 96 hours of exposure to Tregs, stroma, and/or IL-10 neutralizing antibody. The contents of individual wells were normalized to ensure that equal HSC numbers were transplanted. HSCs were retro-orbitally injected into cohorts of sublethally irradiated CD45.1^+^ recipients (550 rads).

### scRNA-seq.

scRNA-seq data (42) was filtered and visualized by graph-based clustering and t-SNE visualization followed by differentially expressed gene and pathway analysis using Partek Flow, version 8.0, Partek Inc.

### Parabiosis Surgery.

Parabiotic surgeries were conducted as previously published by Lever and colleagues (47, 104).

### Murine strains.

C57BL6 mice (stock no. 000664) were obtained from The Jackson Laboratory. Foxp3/GFP mice (stock no. 023800), Foxp3/DTR mice (stock no. 016958), TdTomato mice (stock no. 007909), Ocn-Cre (stock no. 019509), Osx-Cre (stock no. 006361), AdipoQ-Cre (stock no. 010803), and Prrx1-Cre (stock no. 005584) were obtained from The Jackson Laboratory.

Murine strain 10BiT.Foxp3^gfp^ (CD45.1 and CD45.2), immunocompetent, was donated by Casey Weaver (Department of Pathology, University of Alabama at Birmingham, Birmingham, Alabama, USA). Foxp3-Cre/YFP-IL10flox (CD45.2), immunocompetent, was donated by Heth Turnquist (Department of Surgery and Department of Immunology, University of Pittsburgh School of Medicine, Pittsburgh, Pennsylvania, USA).

All murine experiments were conducted using 6- to 12-week-old male and female mice. Mice were separated by sex and housed with 5–7 mice per cage. Littermates of the same sex were randomly assigned to experimental groups. Mice for use in these studies were maintained in a specific pathogen–free state in microisolator caging and received autoclaved water and standard rodent chow diet. Mice were tested quarterly (maximum interval) for all murine pathogens by culture, histopathology, and serology.

### Statistics.

Statistical analyses were reported as mean ± SD or ± SEM. A Shapiro-Wilk test was used to determine normal versus abnormal distributions, and all continuous variables were tested for mean differences. Depending on the spread of variable, both nonparametric Mann-Whitney *U* test, ANOVA, Kruskal-Wallis, and Wilcoxon tests and parametric 2-tailed Student’s *t* test and 1- or 2-way ANOVA were used. For ANOVA, Tukey’s post hoc test was used to compare individual groups (GraphPad Prism version 6.0). A priori sample size calculation was determined based on estimates from preliminary data in order to provide power of > 80% to detect a 30% difference with an α error of 0.05. *P* < 0.05 was considered statistically significant. *P < 0.05, **P < 0.01, ***P < 0.001, ****P < 0.0001.

### Study approval.

All mice were maintained under the guidance of the UAB Animal Resources Program. The Department of Comparative Medicine at the University of Alabama at Birmingham provided veterinary care. UAB complies with the current NIH policy on animal welfare, the Animal Welfare Act, and all applicable federal, state, and local laws. All mice used and the present study was approved by the UAB IACUC (20430).

## Author contributions

VC designed, planned, and performed experiments; analyzed data; and wrote the manuscript. JML, ZY, VRM, SBP, AEL, and PBF performed experiments. LY and HY analyzed data and edited the manuscript. HRT and CLM assisted with data interpretation and experimental design, and edited the manuscript. JFG, ECD, and CTW supervised the study and edited the manuscript. RSW supervised the study and assisted with data interpretation and manuscript writing.

## Supplementary Material

supplemental data

## Version 1 11/19/2020

Electronic publication
