# Supplementary material for: Bone marrow Tregs mediate stromal cell function and support hematopoiesis via IL-10
Source: JCI Insight. 2020 Nov 19;5(22):e135681. doi: 10.1172/jci.insight.135681 (PMC7710301; doi:10.1172/jci.insight.135681)
Supplement: supplemental data [file jciinsight-5-135681-s121.pdf]

Supplement Figures and Data:

A

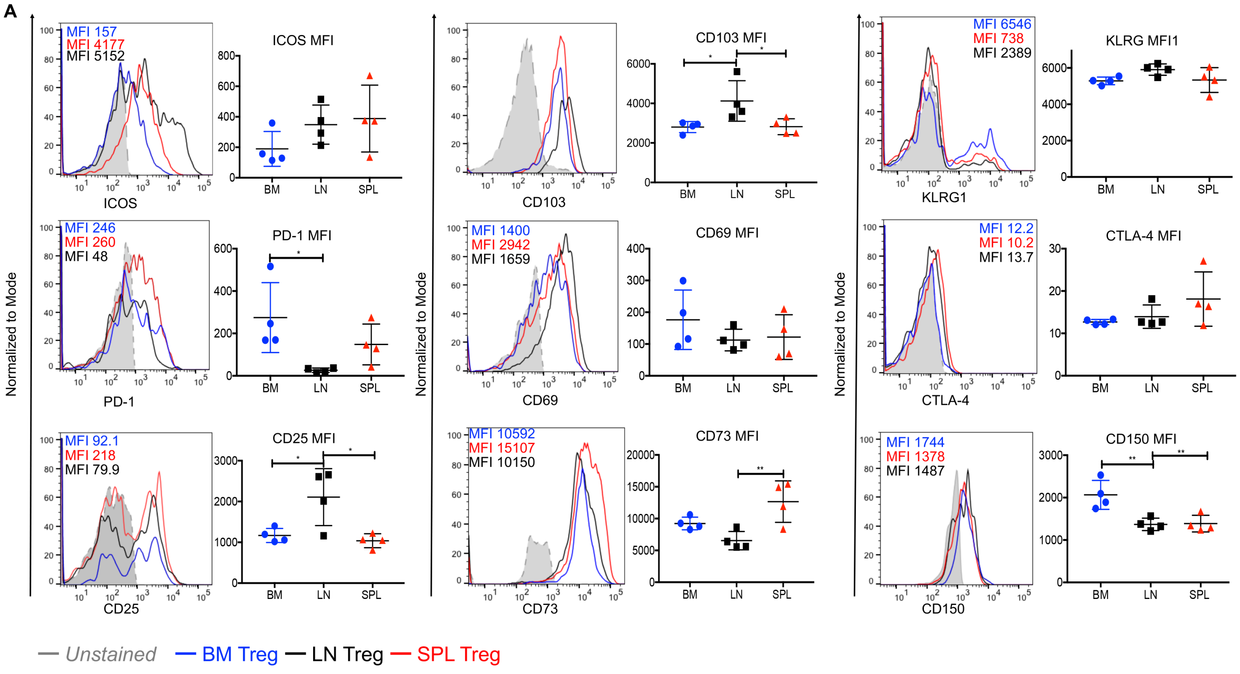

B

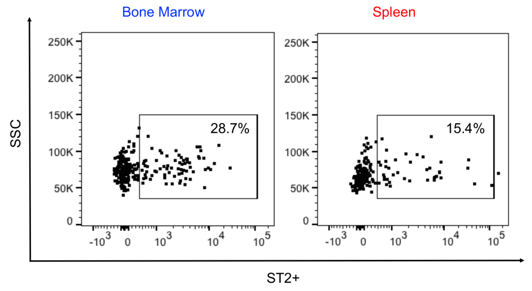

Supplementary Figure 1: Cellular profiling of bone marrow Tregs

S1A. Representative histogram and quantification of MFI of ICOS, CD103, KLRG1, PD-1, CD69, CTLA-4, CD25, CD73 and CD150 in indicated tissues; n=4 animals per group.

S1B. Representative plots of $ST2^{+}$ cells in indicated tissues.

Data are shown as mean ± SD; graphs shown are representative of at least three independent experiments. Statistical analysis performed with one-way ANOVA with Tukey' multiple comparisons test at 95.00% CI of diff (S1C); *p < 0.05, **p < 0.01, ***p < 0.001, ****p < 0.0001.

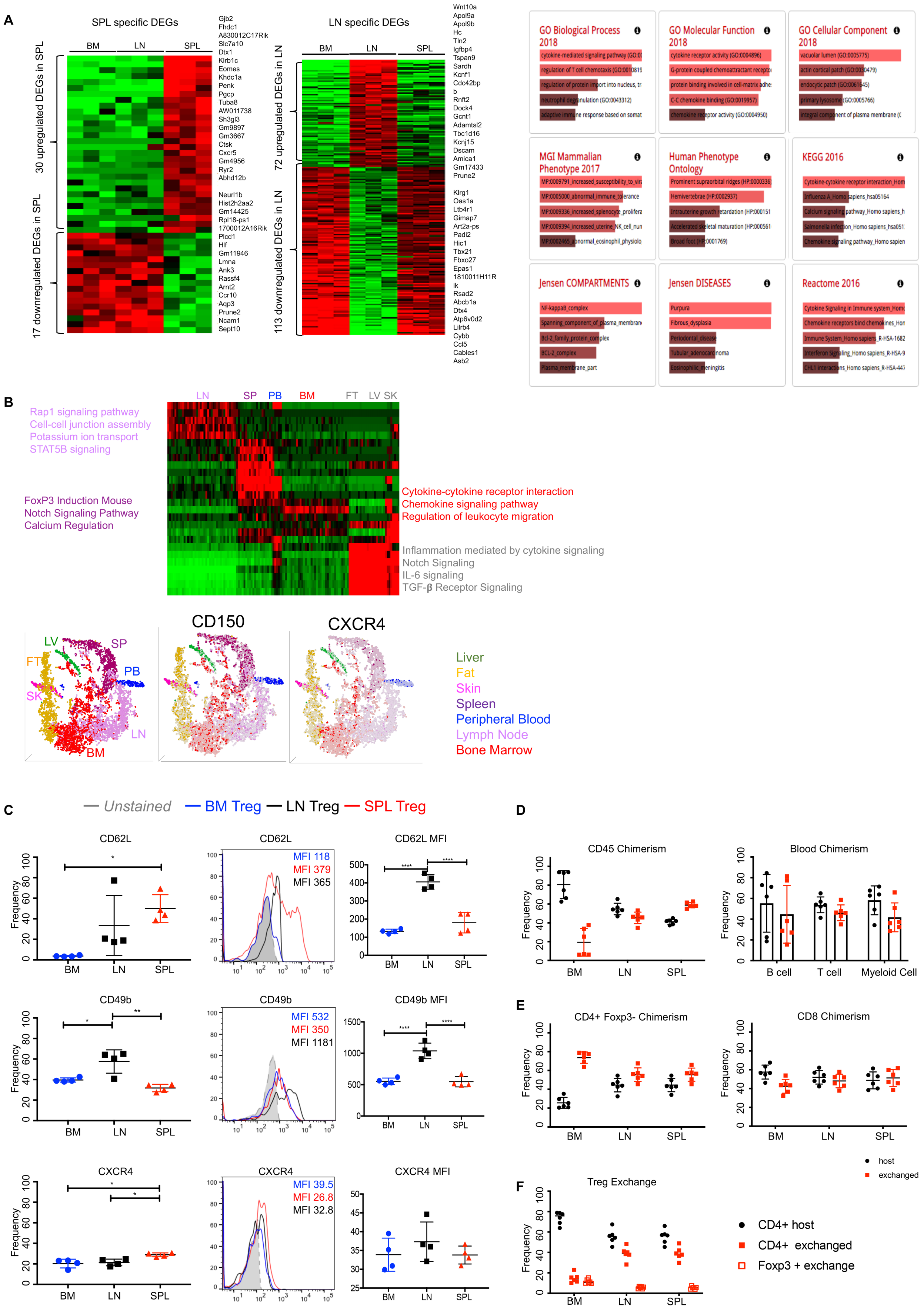

SUPPLEMENTARY FIGURE 2

Supplementary Figure 2: Marrow Tregs localize to their site of origin.

S2A. Heatmap of selected top differentially expressed genes in Foxp3^{+} cells from spleen and lymph node; n=3 biological replicates per tissue sample. Genes with FC with FC< 2 or >2, and adjusted p-value <0.05 are shown (right). GO analysis of enriched pathways in bone marrow Foxp3^{+} cells; n=3 biological replicates (left).

S2B. Heatmap of top differentially expressed pathways from single-cell transcriptomes of tissue Treg populations: lymph (LN), spleen (SP), peripheral blood (PB), bone marrow (BM), fat (FT), and skin (SK) Tregs (right). Relative abundance of CD150 and CXCR4 expression among tissue Treg populations is shown (bottom).

S2C. Frequencies and representative MFI of Foxp3^{+}cells showing frequencies and representative MFI histograms of CD62L, CD49b, and CXCR4 from bone marrow (blue) lymph node (black) spleen (red) and unstained controls.

S2D: Cellular exchange in bone marrow, lymph node, and spleen of parabionts (left). Exchange of B cell, T cell, and Myeloid cell lineages in peripheral blood (right). Host (black) and donor populations (red).

S2E: Total chimerism of CD4^{+} (left) and CD8^{+} cells (right) in bone marrow lymph node and spleen of parabionts. Host (black) and donor populations (red).

S2F: Frequency of Treg exchange among CD4^{+} cells in bone marrow, lymph node, and spleen of parabionts; Foxp3^{+}CD4^{+} exchanged (red), Foxp3^{-}CD4^{+} exchanged (red), Foxp3^{-}CD4^{+} resident (black).

Data are shown as mean ± SD; graphs shown are representative of at least three independent experiments. Statistical analysis performed with one-way ANOVA with Tukey' multiple comparisons test at 95.00% CI of diff (S2C); *p < 0.05, **p < 0.01, ***p < 0.001, ****p < 0.0001.

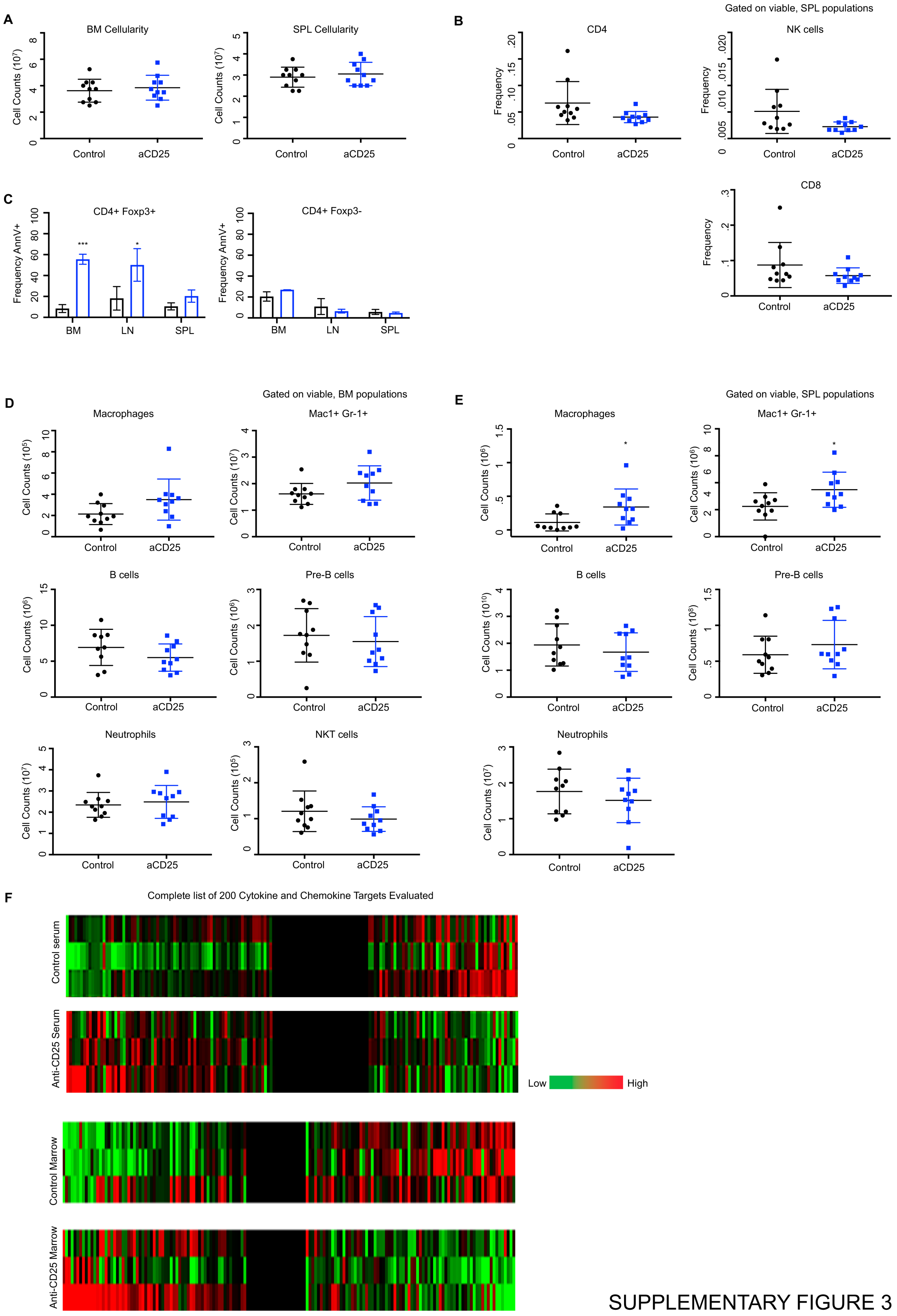

SUPPLEMENTARY FIGURE 3

Supplementary Figure 3: Treg depletion does not result in inflammation

S3A: Absolute counts of bone marrow and spleen cellularity following anti-CD25; control (black), anti-CD25 (blue); n=10 animals per group for each tissue.

S3B: Absolute frequencies of $CD4^+$, $CD8^+$ and $NK1.1^+$ cells following anti-CD25; control (black), anti-CD25 (blue); n=10 animals per group.

S3C. Frequency of $AnnexinV^+$ cells in $Foxp3^+CD4^+$ and $Foxp3^-CD4^+$ cells following anti-CD25; control (black), anti-CD25 (blue) (left); n=4 animals per group.

S3D. Absolute counts of bone marrow populations following anti-CD25: Macrophages, Myeloid cells, B cells, Neutrophils, and NKT cells; control (black), anti-CD25 (blue) n=10 animals per group.

S3E. Absolute counts of splenic populations following anti-CD25: Macrophages, Myeloid cells, B cells, Neutrophils; control (black), anti-CD25 (blue); n=10 animals per group.

F. Complete target list of serum and bone marrow fluid cytokines and chemokines following anti-CD25 depletion (ordered based on fold change).

Data are shown as mean ± SD; graphs shown are representative of at least three independent experiments. Statistical analysis performed with unpaired two-tailed Student *t* test (3A-E). *$p < 0.05$, **$p < 0.01$, ***$p < 0.001$, ****$p < 0.0001$.

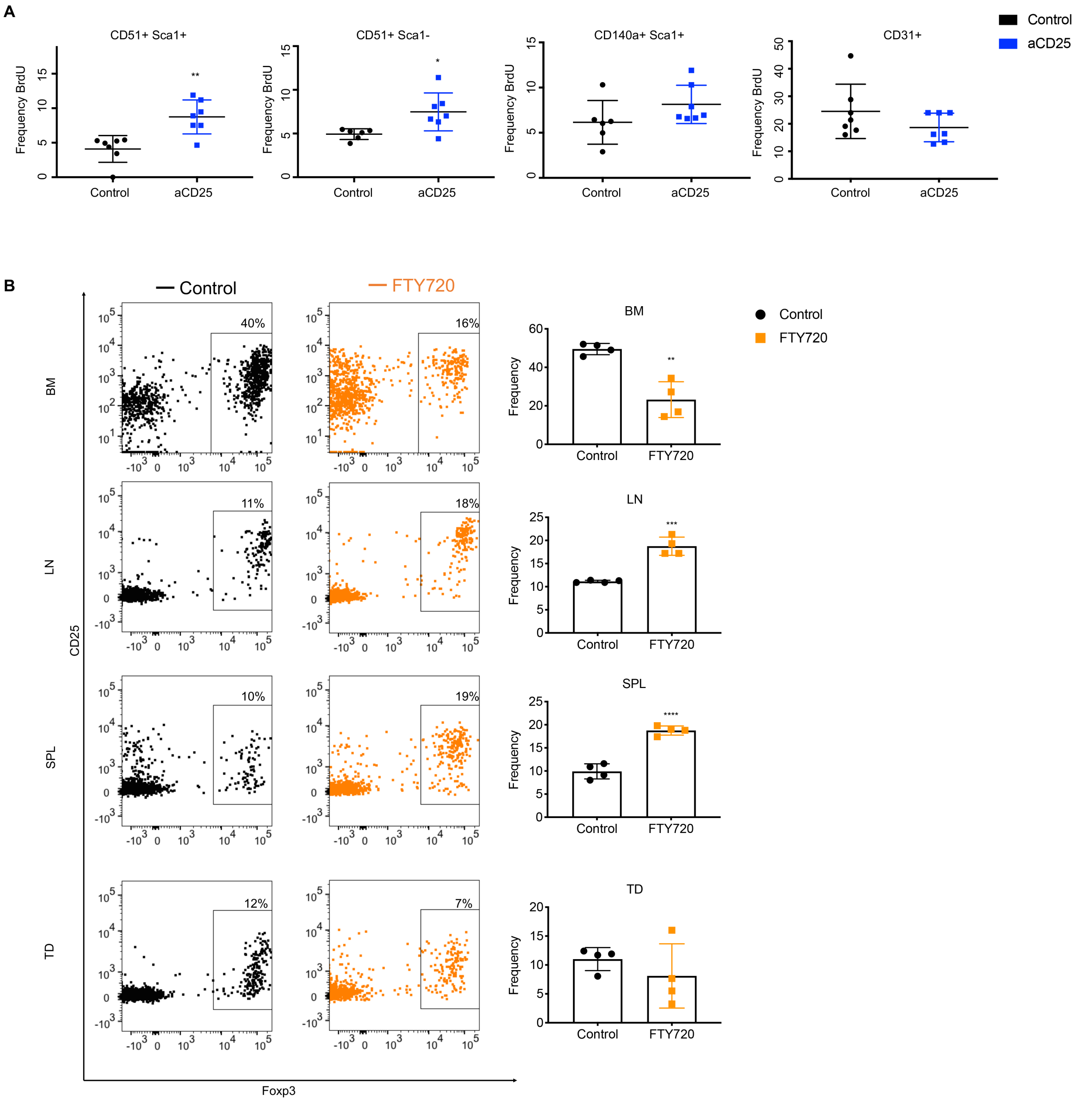

SUPPLEMENTARY FIGURE 4

Supplementary Figure 4: Tregs are required for stromal cell maintenance
S4A. Frequency of BrdU^{+} cells: CD51^{+}Sca1^{+}cells (MSCs), CD51^{+} Sca1^{-} (bone precursors), CD140α^{+} Sca-1^{+}(PDGFRα^{+} cells), endothelial cells (CD31^{+}) following anti-CD25; control (black), anti-CD25 (blue); n=7 animals per group.
S4B: Representative plot of Foxp3^{+} cells in bone marrow, lymph node, spleen and thoracic duct (right); frequencies of Foxp3^{+} cells (left); control (black), FTY720 (orange); n=4 animals per group.
Data are shown as mean ± SD; graphs shown are representative of at least three independent experiments. Statistical analysis performed with unpaired two-tailed Student *t* test (S4A-B)*$p < 0.05$, **$p < 0.01$, ***$p < 0.001$, ****$p < 0.0001$.

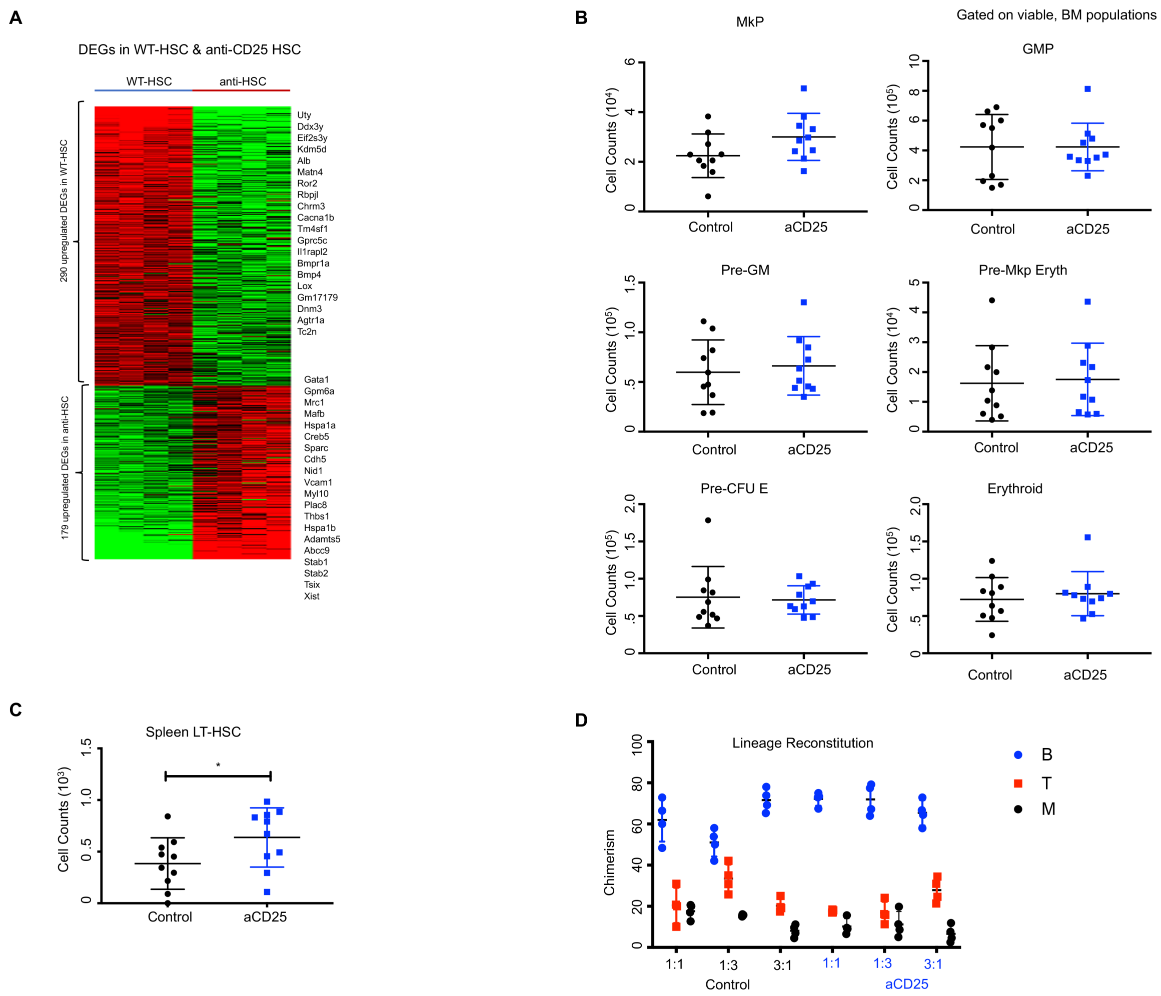

SUPPLEMENTARY FIGURE 5

Supplementary Figure 5: Bone marrow Tregs enhance HSC-supportive activity of stromal cells

S5A. Heatmap of top differentially expressed genes in LT-HSC's from control and anti-CD25 mice; n=4 biological replicates per treatment condition. Genes with FC< 1/2 or >2, and adjusted p-value <0.05 are shown.

S5B. Absolute counts of bone marrow populations following anti-CD25: MkP, GMP, pre-GM, Pre Mkp Eryth, Pre-CFU-E, Erythroid; control (black), anti-CD25 (blue); n=10 animals per group.

S5C. Absolute counts of splenic LT-HSC populations following anti-CD25; control (black), anti-CD25 (blue); n=10 animals per group.

S5D. Multi-lineage engraftment of B cell (blue); T cell (red) and Myeloid cell (black) lineages; control (black), anti-CD25 (blue); n=4 recipients per group.

Data are shown as mean ± SD; graphs shown are representative of at least three independent experiments. Statistical analysis performed with unpaired two-tailed Student *t* test (4A-C) *p < 0.05, **p < 0.01, ***p < 0.001, ****p < 0.0001.

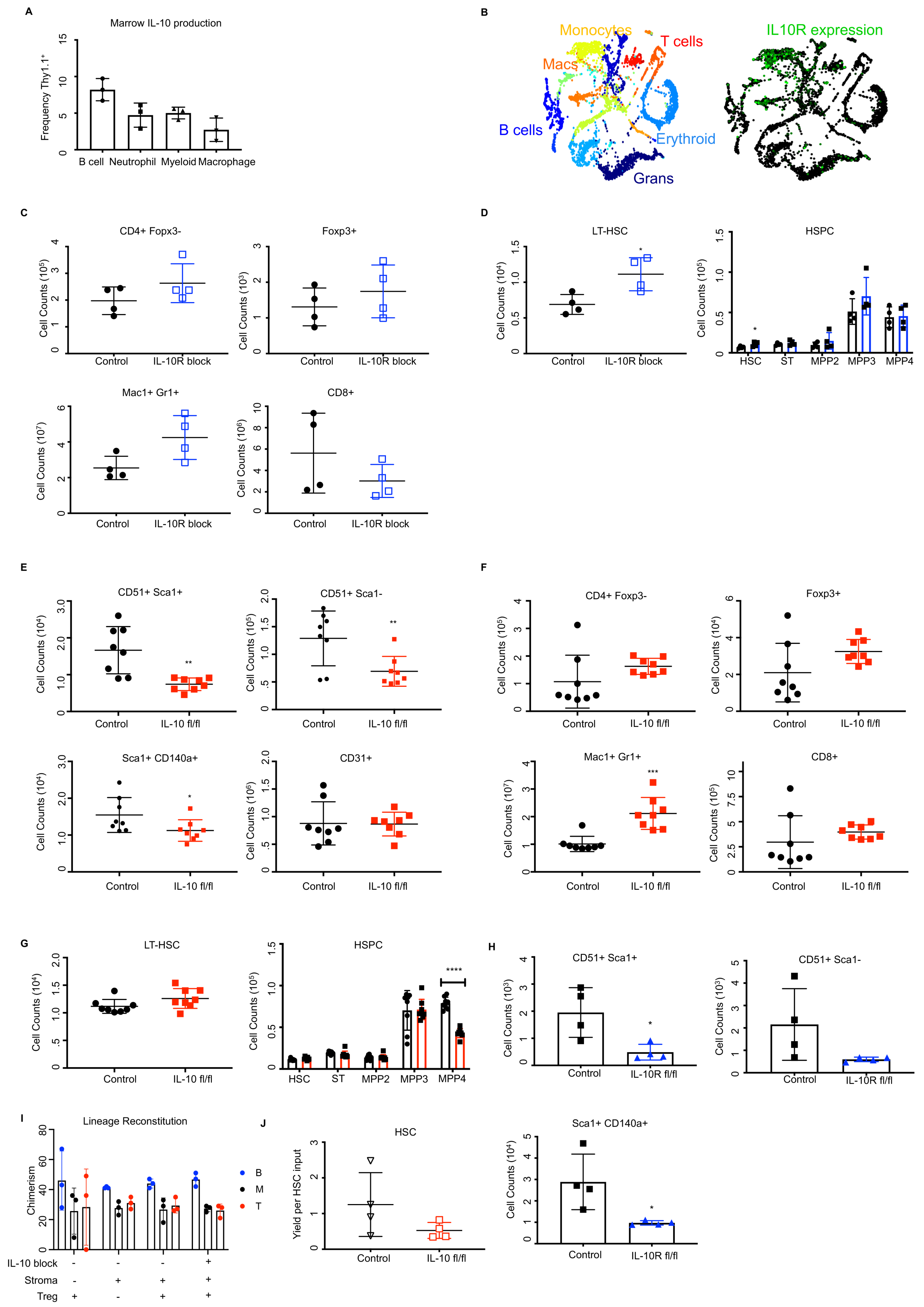

SUPPLEMENTARY FIGURE 6

Supplementary Figure 6: Treg IL-10 restricts stromal cell proliferation and differentiation

S6A: Quantification of IL-10-expressing (Thy1.1^{+}) cells in B cell, Neutrophil, Myeloid cells, Macrophages; n=3 animals per group.

S6B: SPRING plots of mature bone marrow populations: B cell, Neutrophil, Myeloid cells, Macrophages (right). Relative abundance of IL-10Ra expression within bone marrow populations is shown in green (left).

S6C. Absolute counts of Foxp3^{-} CD4^{+}, Foxp3^{+}CD4^{+}, CD8^{+}, and Mac1^{+}Gr1^{+} cells in IL-10R^{BLOCK} mice; control (black), IL-10R^{BLOCK} (blue); n=4 animals per group.

S6D. Absolute counts of HSPCs in IL-10R^{BLOCK} mice; control (black), IL-10R^{BLOCK} (blue); n=4 animals per group.

S6E. Absolute counts of CD51^{+} Sca1^{+}cells (MSCs), CD51^{+}Sca1^{-}(bone precursors), and CD140α^{+}Sca-1^{+} (PDGFRα^{+} cells), endothelial cells (CD45^{-}/Ter119^{-}CD31^{+}) in Foxp3-Cre×IL-10$^{fl/fl}$ mice; control (black),10$^{fl/fl}$ (red); n=8 animals per group.

S6F. Absolute counts of Foxp3^{-} CD4^{+}, Foxp3^{+}CD4^{+}, CD8^{+}, and Mac1^{+}Gr1^{+} cells in Foxp3-Cre×IL-10$^{fl/fl}$ mice; control (black),10$^{fl/fl}$ (red); n=8 animals per group.

S6G. Absolute counts of HSPCs in Foxp3-Cre×IL-10$^{fl/fl}$ mice; control (black),10$^{fl/fl}$ (red); n=8 animals per group.

S6H. Absolute counts of CD51^{+}Sca1^{+}cells (MSCs), and CD31^{-}CD51^{+} Sca1^{-} bone precursors, and CD140α^{+}Sca-1^{+} (PDGFRα $^{+}$), in Prrx1-Cre×IL10Rα$^{fl/fl}$ mice; control (black),10Rα$^{fl/fl}$ (blue) n=4 animals per group.

S6I. Multi-lineage engraftment of ex-vivo cultured HSC: B cell (blue); T cell (red) and Myeloid cell (black); n=3 recipients per group.

S6J. Co-culture of LT-HSCs maintained on WT stroma (black) or Foxp3-Cre×IL-10$^{fl/fl}$ stroma (red) for 96 hours; Data expressed as fold-change relative to input of HSCs; n=4 wells per condition.

Data are shown as mean ± SD; graphs shown are representative of at least three independent experiments. Statistical analysis performed with unpaired two-tailed Student *t* test (6C-G). *$p < 0.05$, **$p < 0.01$, ***$p < 0.001$, ****$p < 0.0001$

Supplemental Table 1: Complete Antibody Information.

Antibody	Clones	Catalog Number	Source
KLRG1	14C2A07	138411	Biolegend
ICOS	15F9	117405	Biolegend
CD25	PC67	102049	Biolegend
CD103	2E7	121423	Biolegend
CD44	IM7	103028	Biolegend
CD62L	MEL-14	104410	Biolegend
CD49b	DX5	108909	Biolegend
CD127	A7R34	135010	Biolegend
CTLA-4	UC10-4B9	17-1522-82	eBioscience
PD-1	RMP1-14	114117	Biolegend
CD73	AD2, TY/11.8	563198, 127210	Fisher Scientific, Biolegend
CD69	FN50	310911	Biolegend
CXCR4	12G5	306509	Biolegend
CD150	TC15	115941	Biolegend
S1PR1	SW4GYPP	50-3639-41	ThermoFisher Scientific
ST2	U29-93	U29-93	BD Biosciences
CD3	145-2C11, 17A2	100351, 100222	Biolegend
CD4	GK1.5	100426	Biolegend
CD8	53-6.7	100714	Biolegend
NK-1.1	PK136	108719	Biolegend
Ly-6G/Ly-6C (Gr-1)	RB6	108410	Biolegend
CD115	AFS98	135510	Biolegend
CD11b	M1/70	101204	Biolegend
F4/80	BM8	123118	Biolegend
CD45R/B220	RA3-6B2	103206	Biolegend
CD19	6D5	115510	Biolegend
CD43	S11	143204	Biolegend
Ter119	Ly-76	116233	Biolegend
Flk-2, Flt3	A2F10	135315	Biolegend
CD34	RAM34	11-0341-82	Biolegend
CD48	HM48-1	103439	Biolegend
CD45	30-F11	103110	Biolegend
Ly-6A/E (Sca-1)	D7	108134	Biolegend
CD31	390	102422	Biolegend
c-kit (CD117)	2B8	105826	Biolegend
CD140a	APA5	25-1401-80	eBioscience
CD51	RMV-7	104106	Biolegend
CD45.2	104	109806	Biolegend
CD45.1	A20	110741	Biolegend
CD41	MWReg30	133904	Biolegend
